# Supplementary figures and images for: Does deep neuromuscular blockade provide improved perioperative outcomes in adult patients? A systematic review and meta-analysis of randomized controlled trials
Source: PLoS One. 2023 Mar 9;18(3):e0282790. doi: 10.1371/journal.pone.0282790 (PMC9997990; doi:10.1371/journal.pone.0282790)

(A)

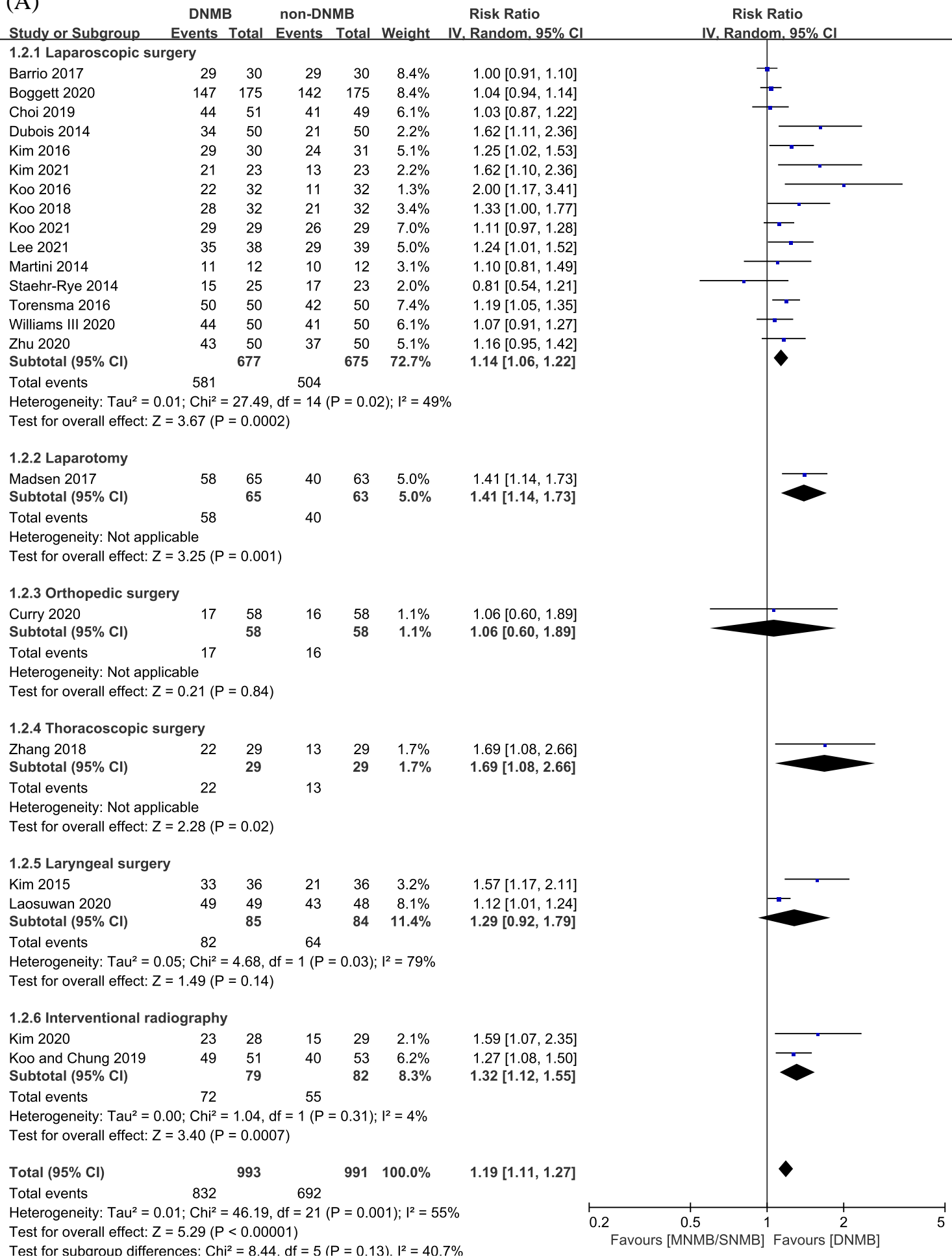

(B)

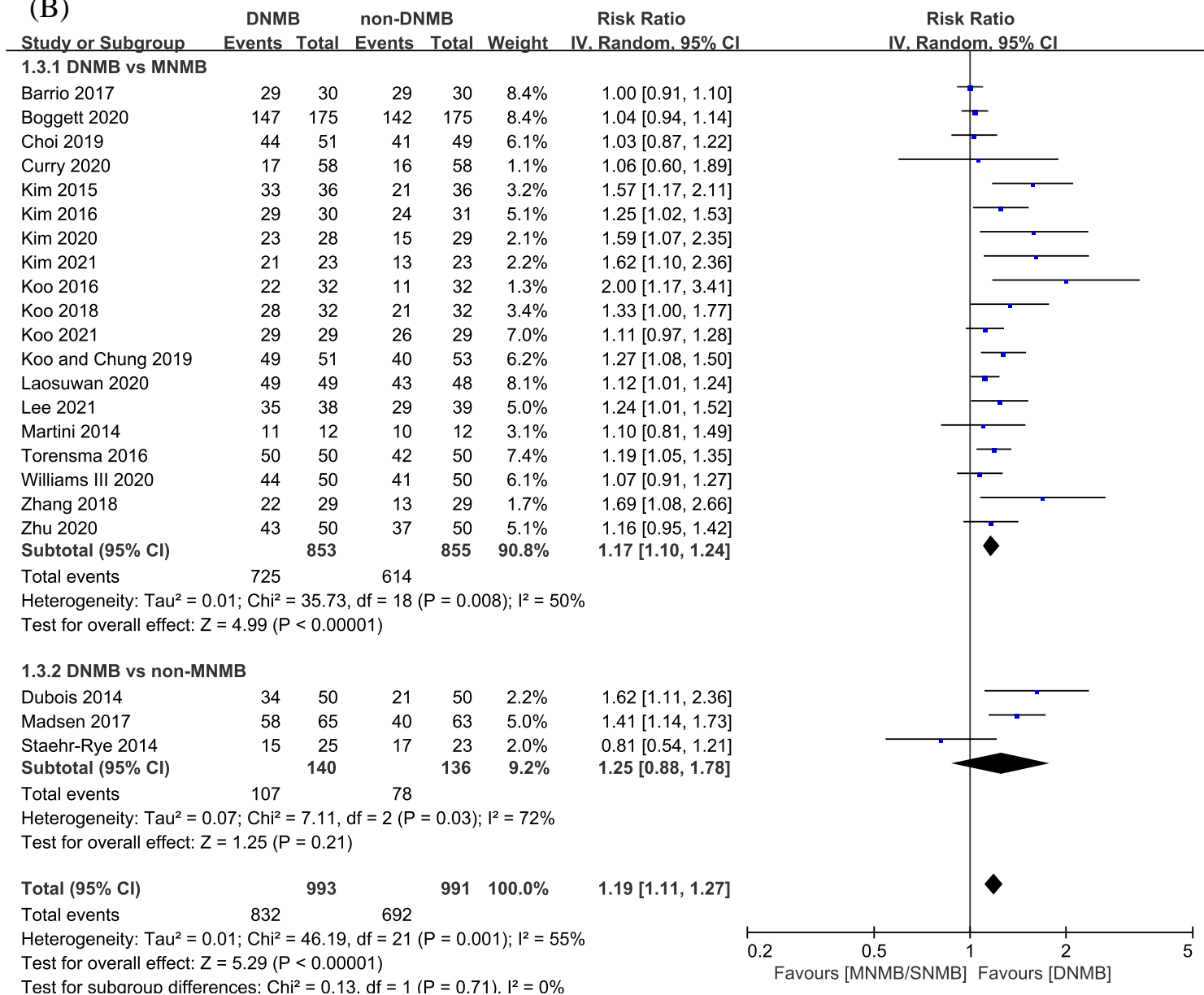

(C)

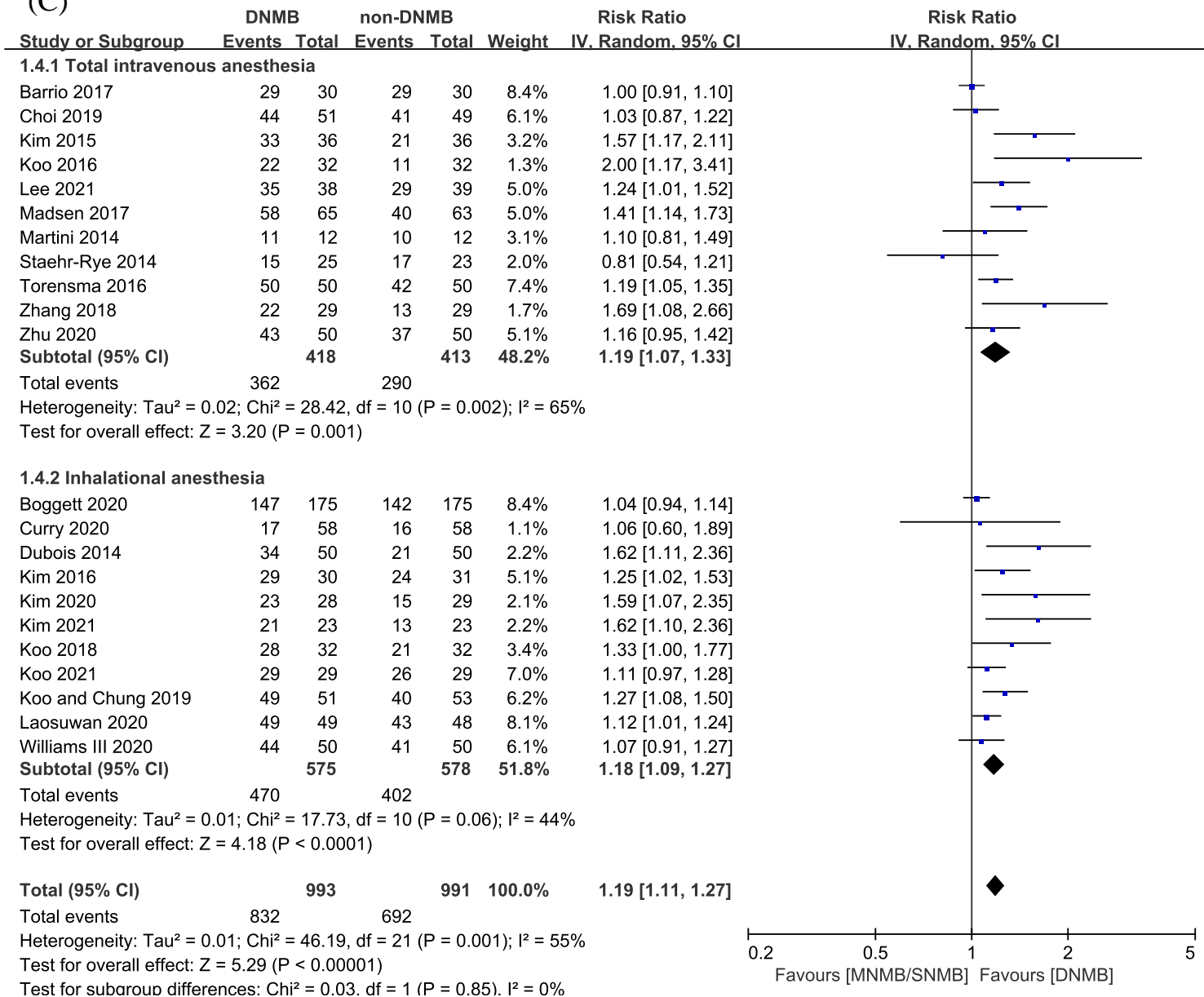

Supplement: S1 Fig — (A) Type of surgery, (B) Depth of neuromuscular blockade, (C) Type of anesthesia. (PDF) [file pone.0282790.s001.pdf]

(A)

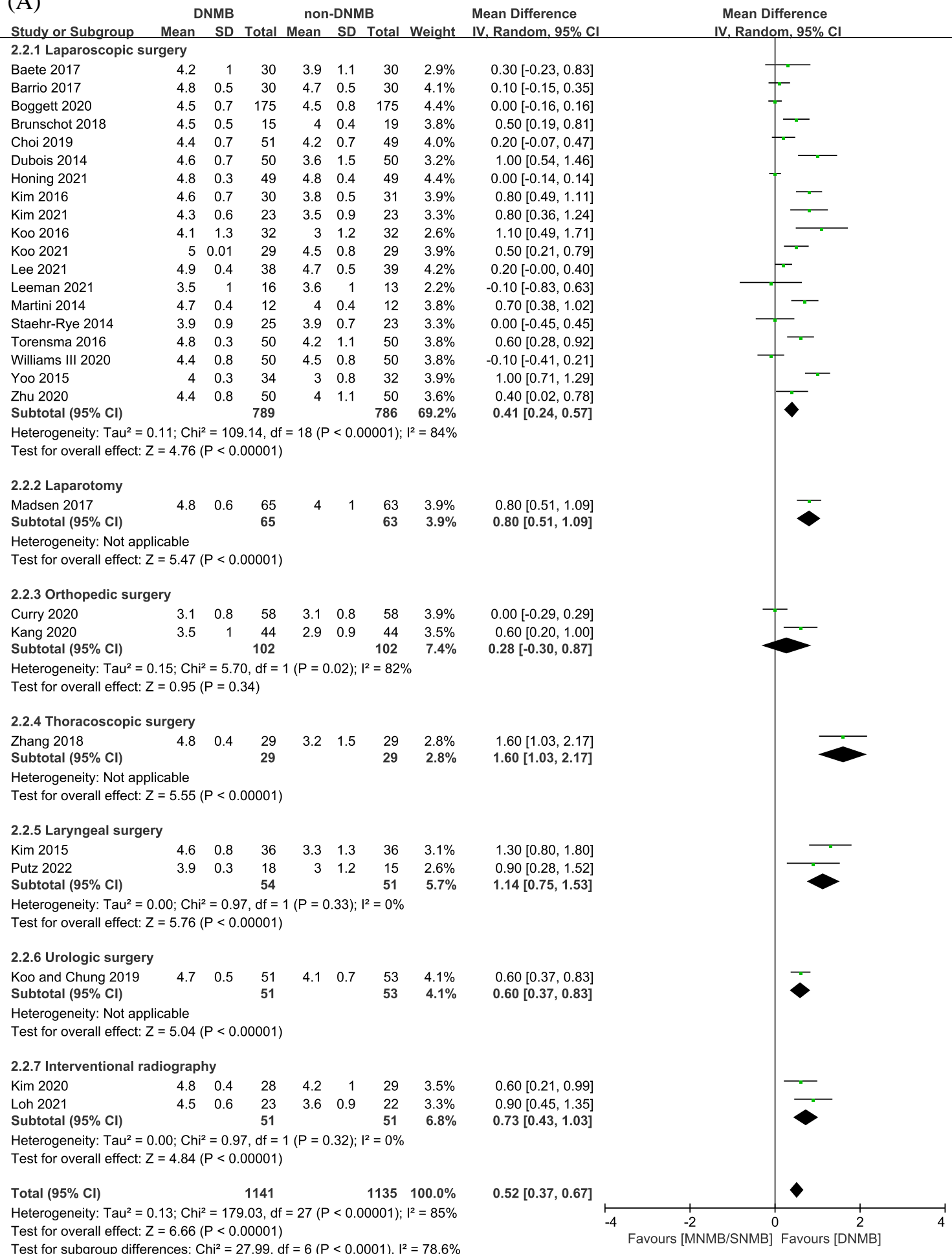

(B)

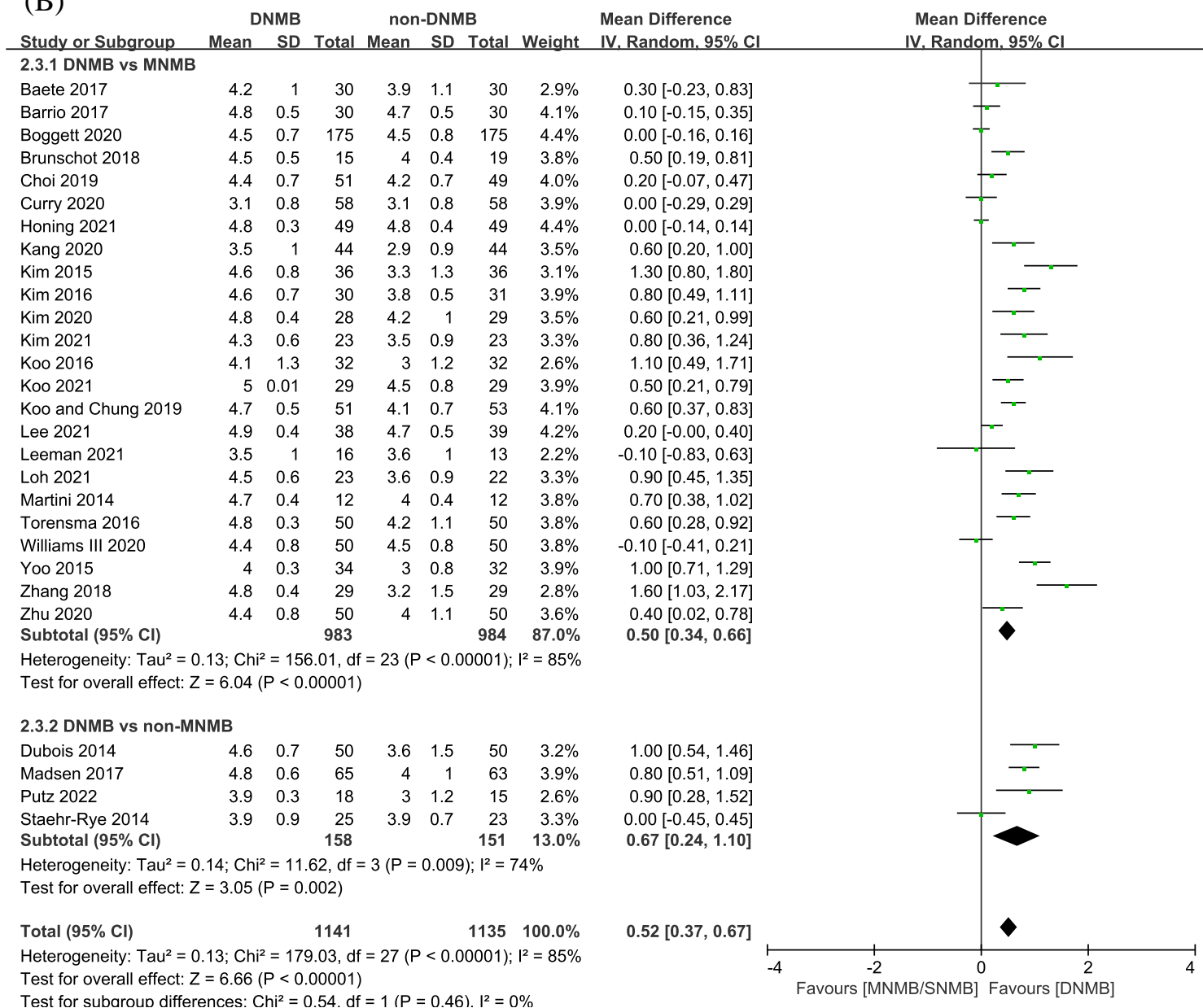

(C)

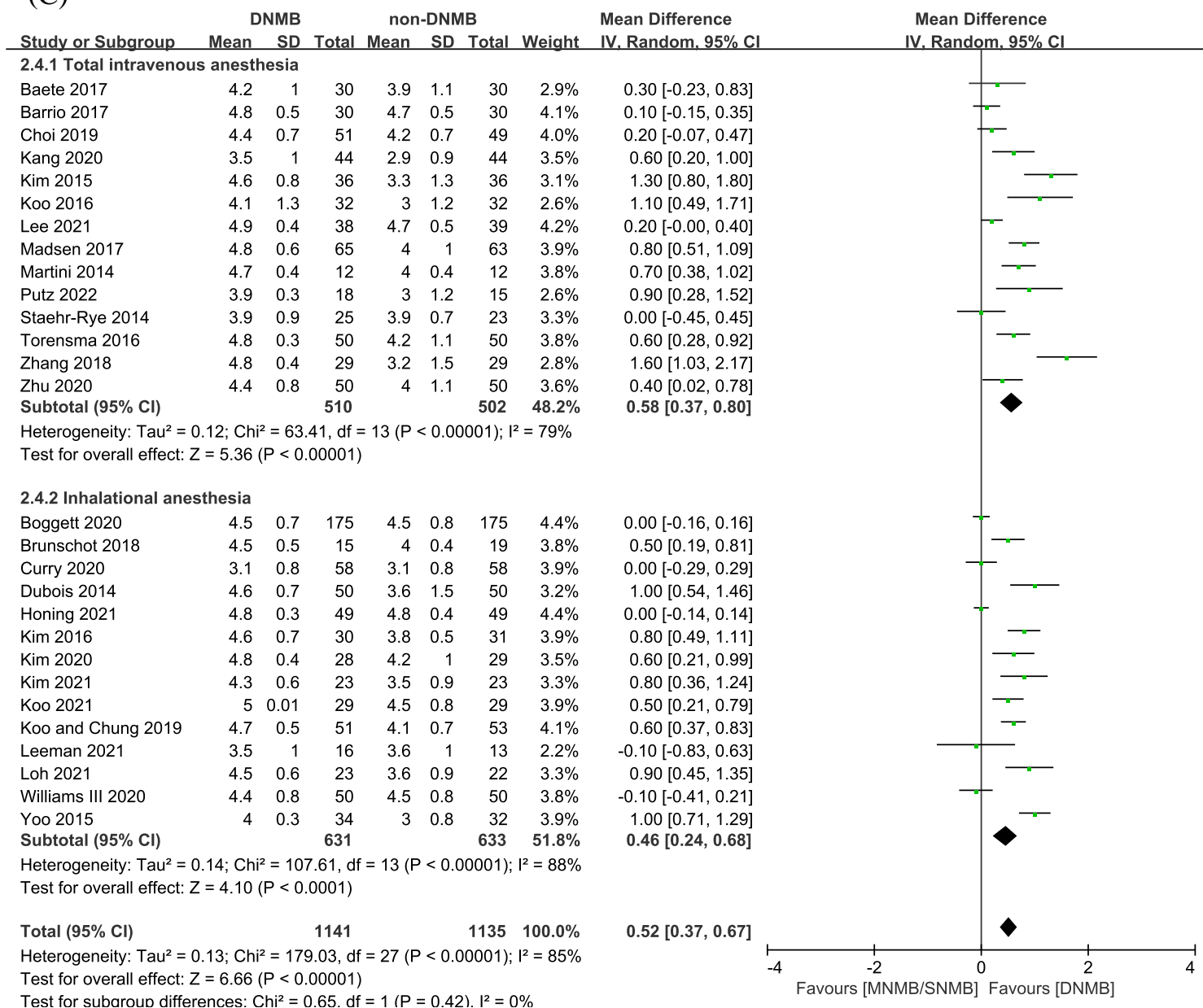

Supplement: S2 Fig — (A) Type of surgery, (B) Depth of neuromuscular blockade, (C) Type of anesthesia. (PDF) [file pone.0282790.s002.pdf]

(A)

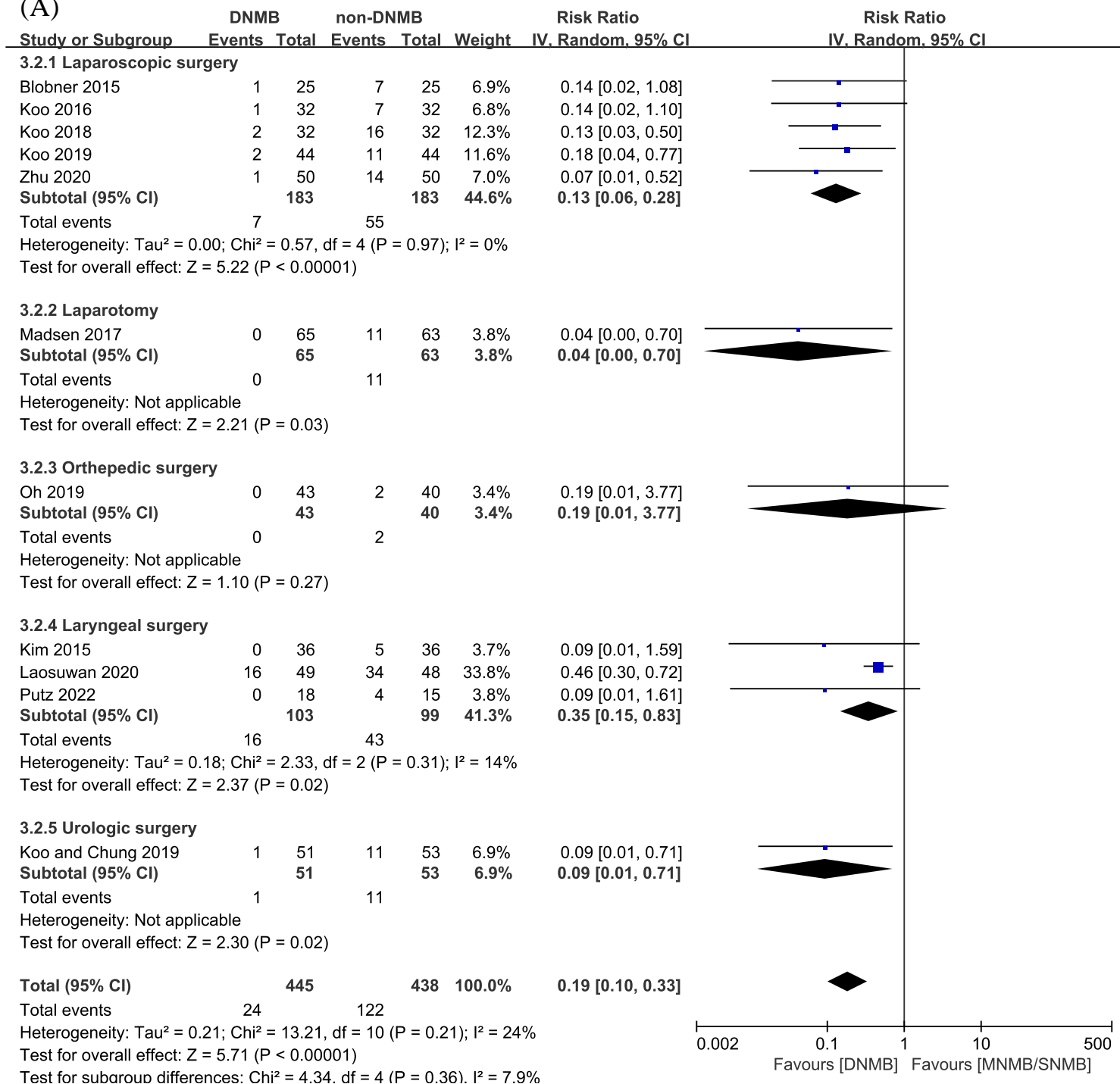

(B)

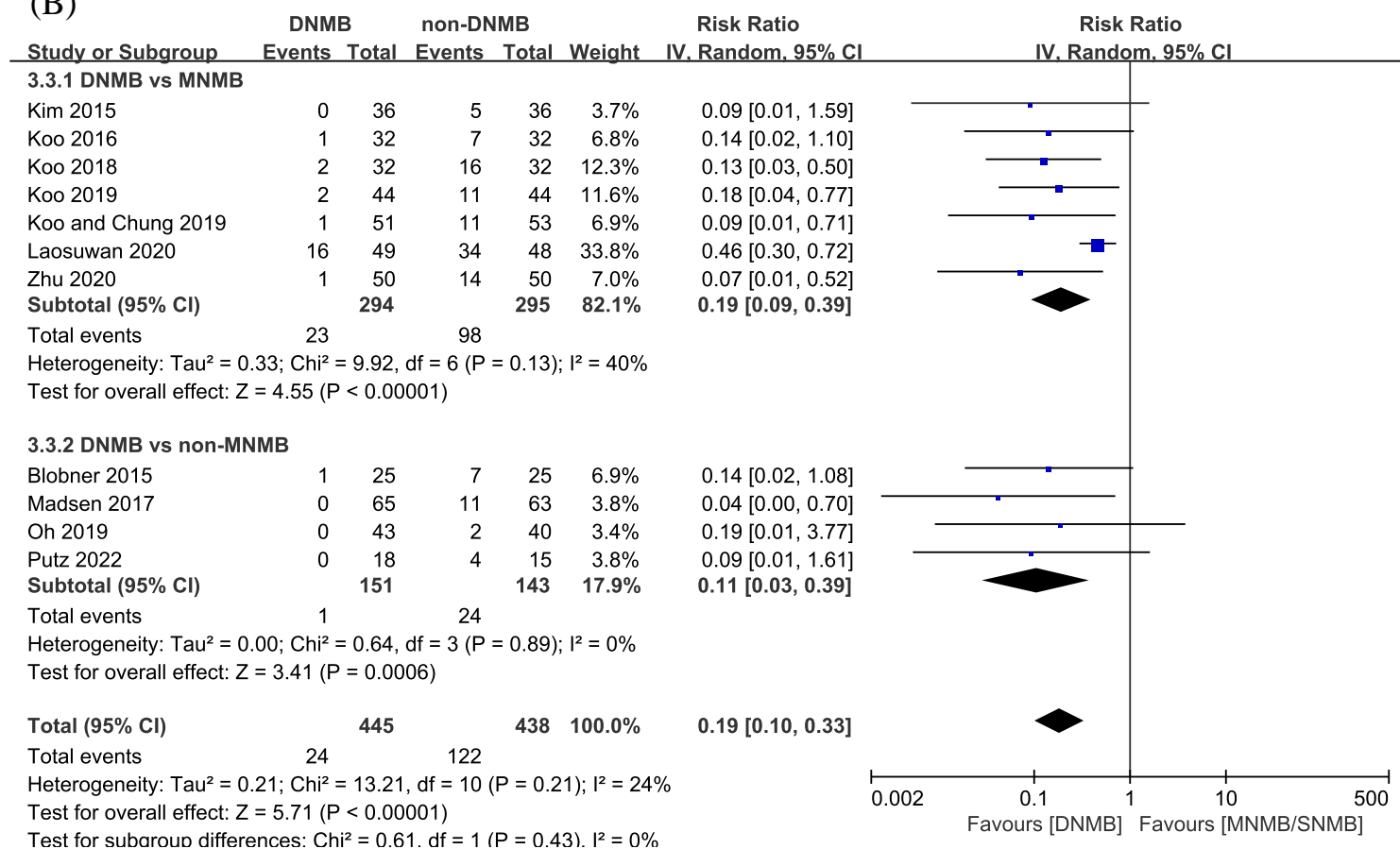

(C)

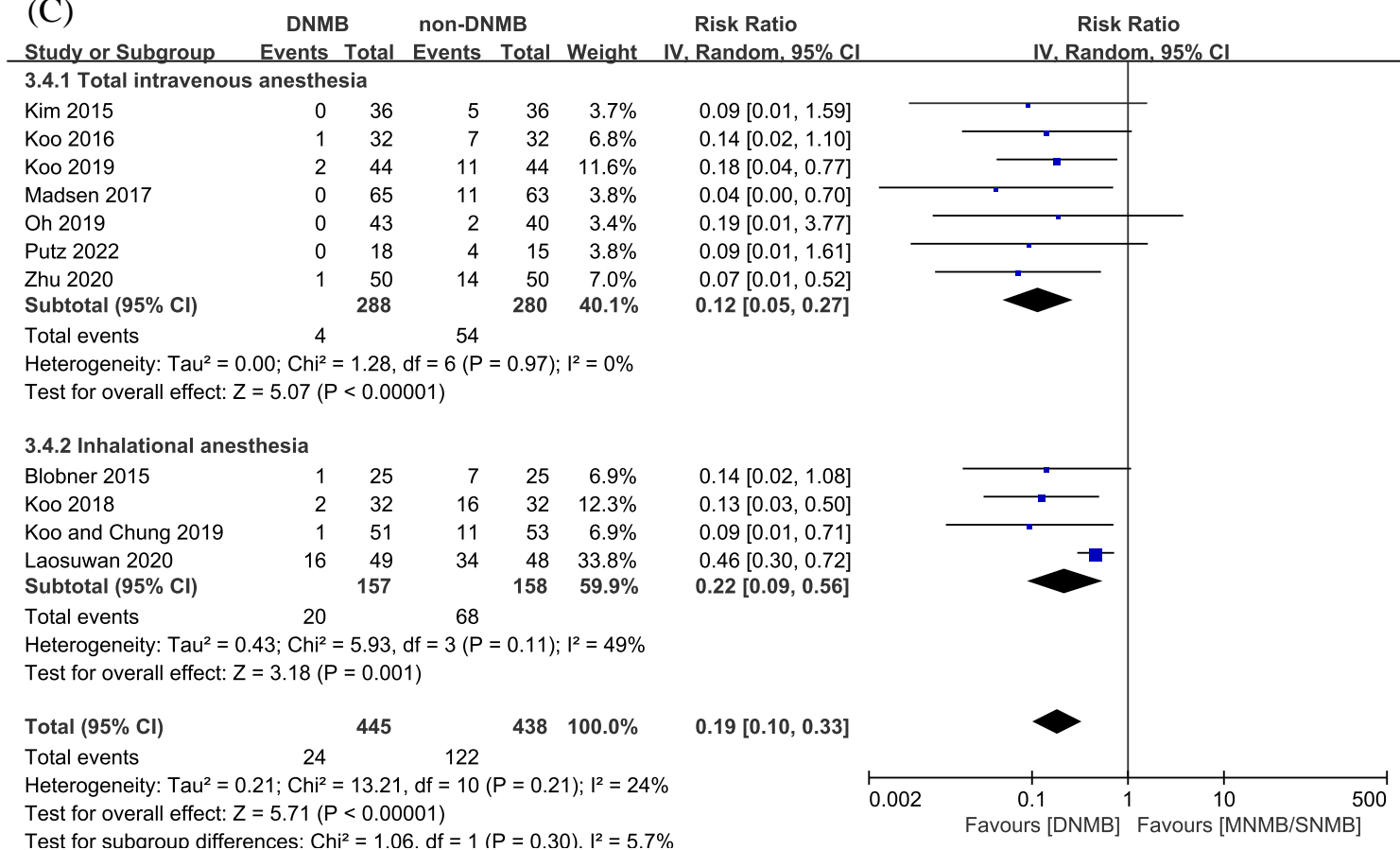

Supplement: S3 Fig — (A) Type of surgery, (B) Depth of neuromuscular blockade, (C) Type of anesthesia. (PDF) [file pone.0282790.s003.pdf]

(A)

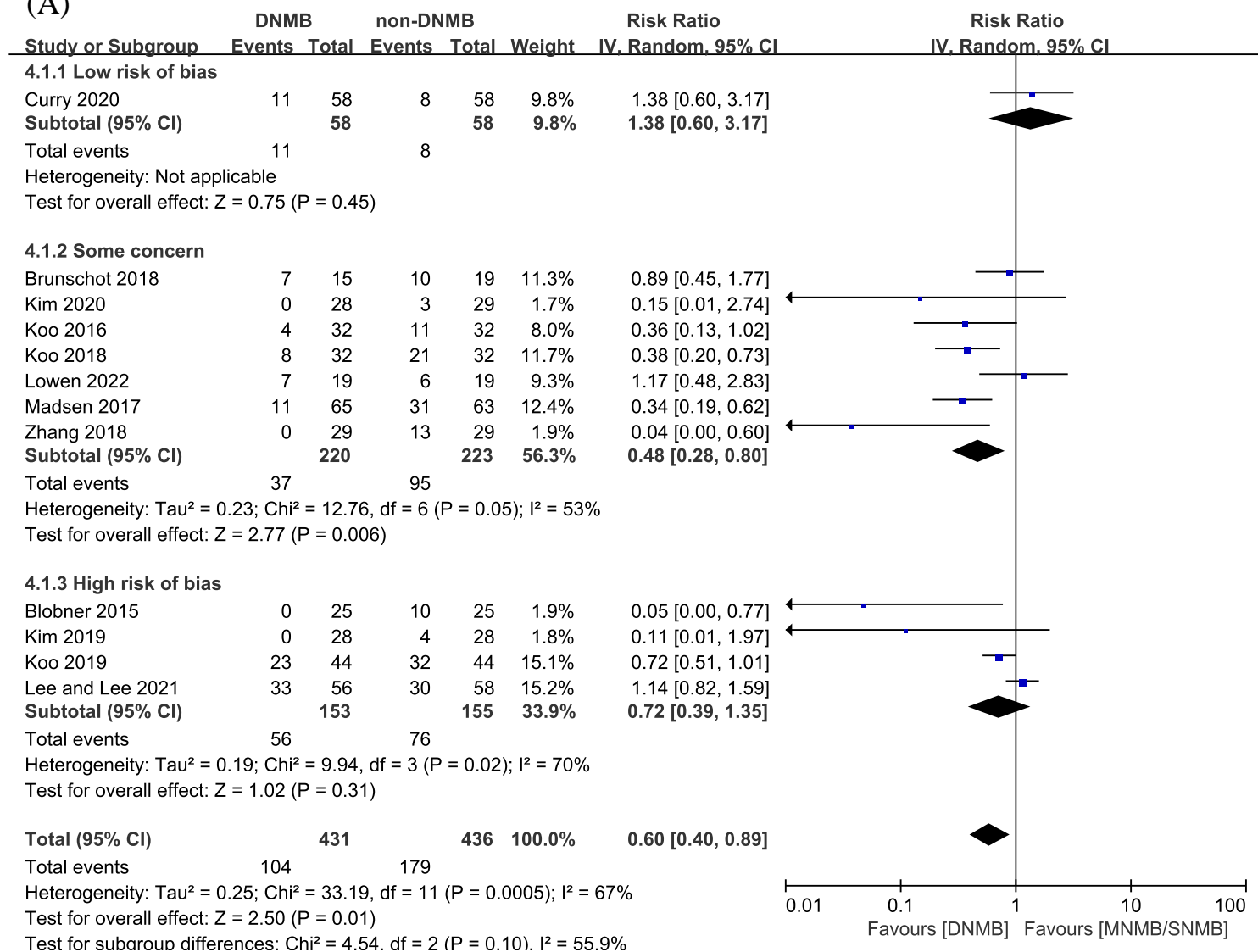

(B)

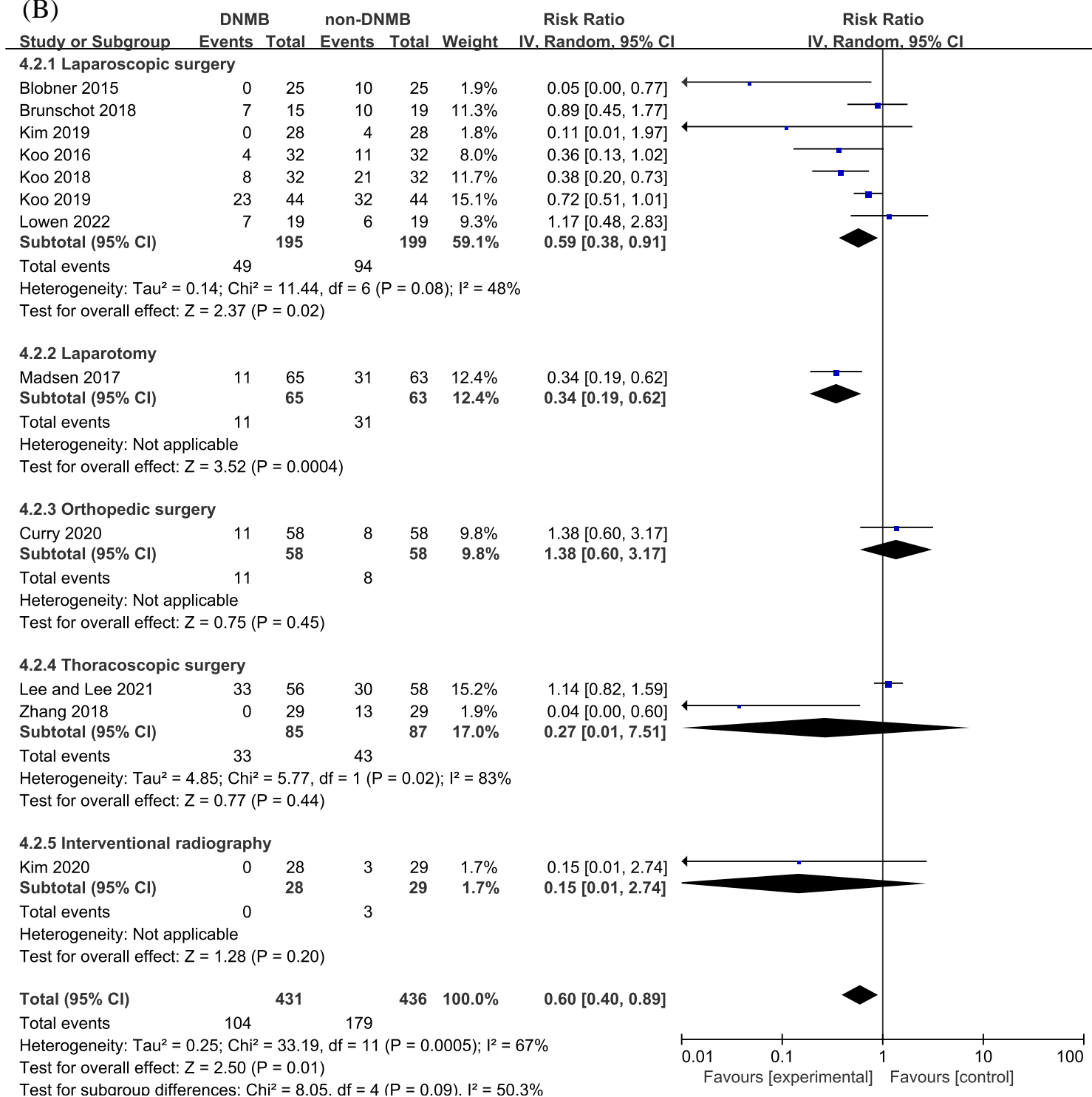

(C)

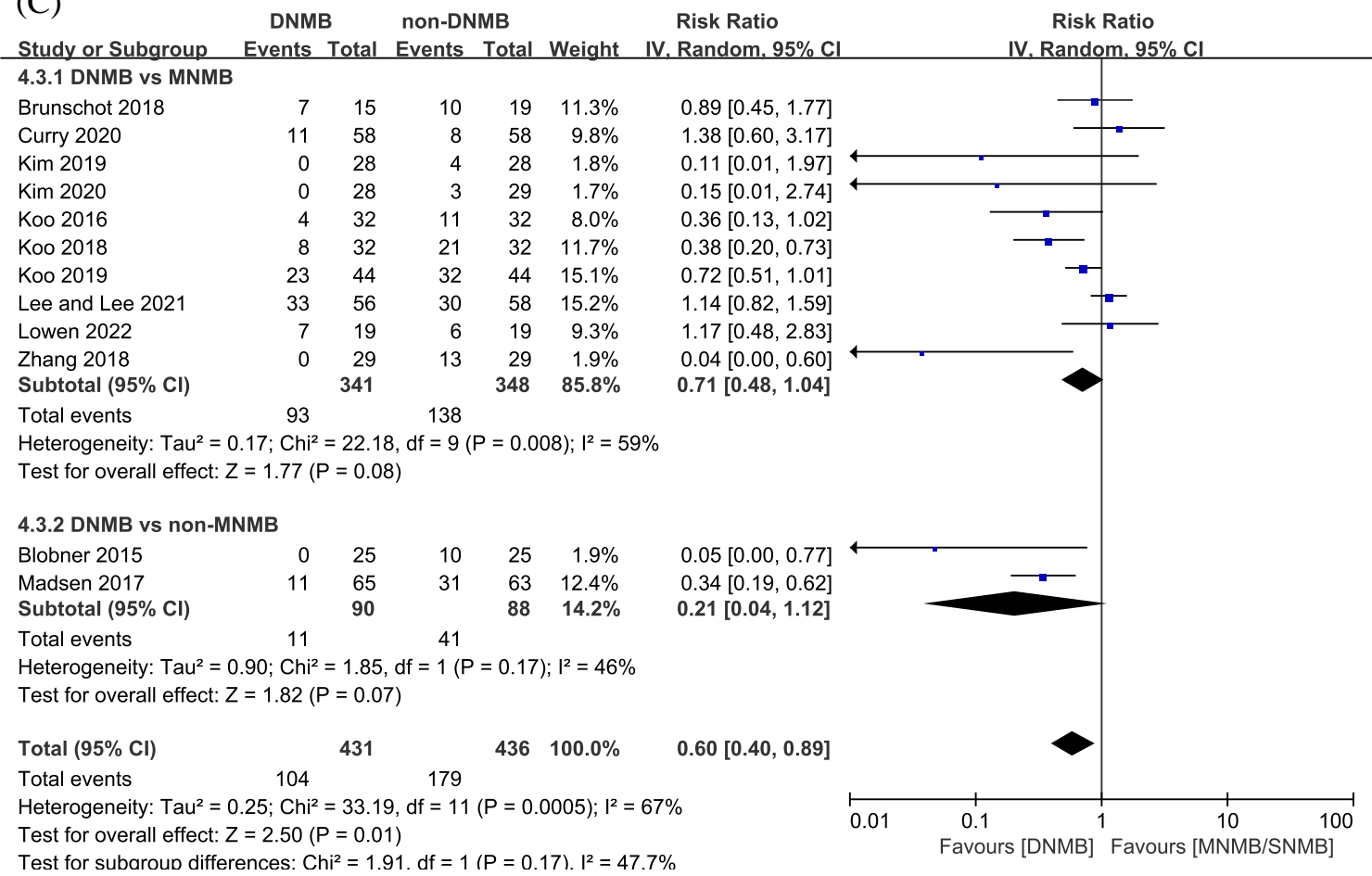

(D)

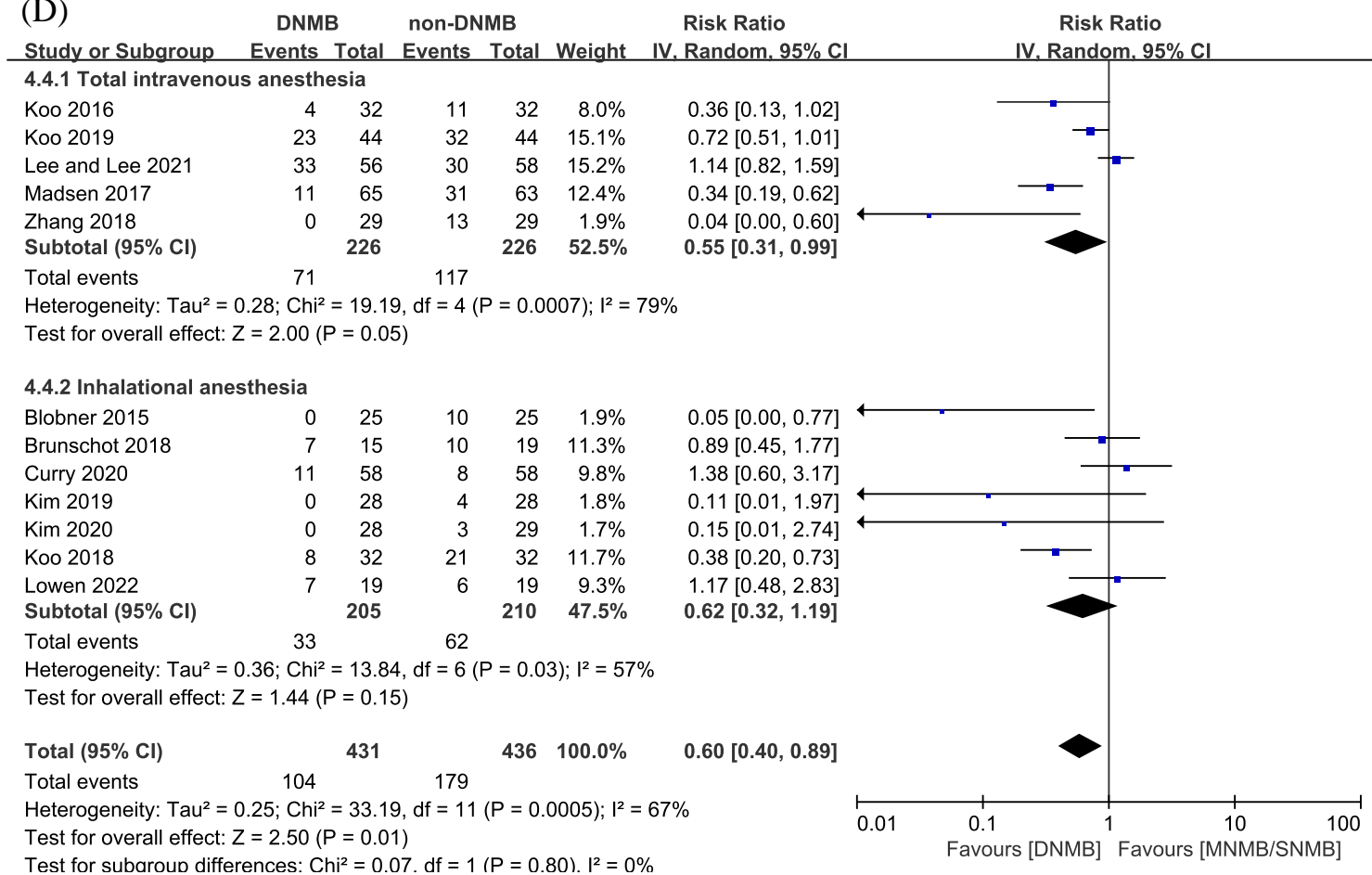

Supplement: S4 Fig — (A) Primary analysis, (B) Subgroup based on the type of surgery, (C) Subgroup based on the depth of neuromuscular blockade, (D) Subgroup based on the type of anesthesia. (PDF) [file pone.0282790.s004.pdf]

(A)

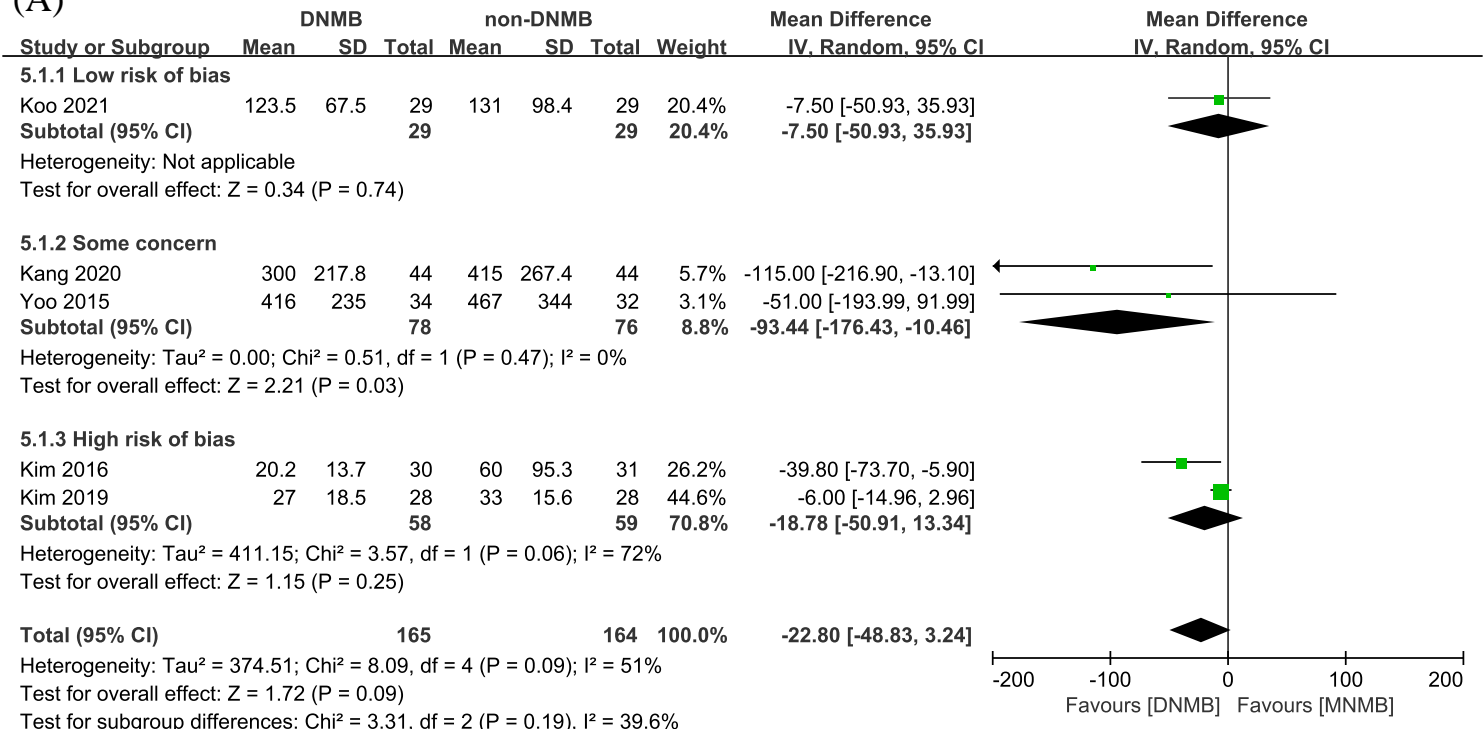

(B)

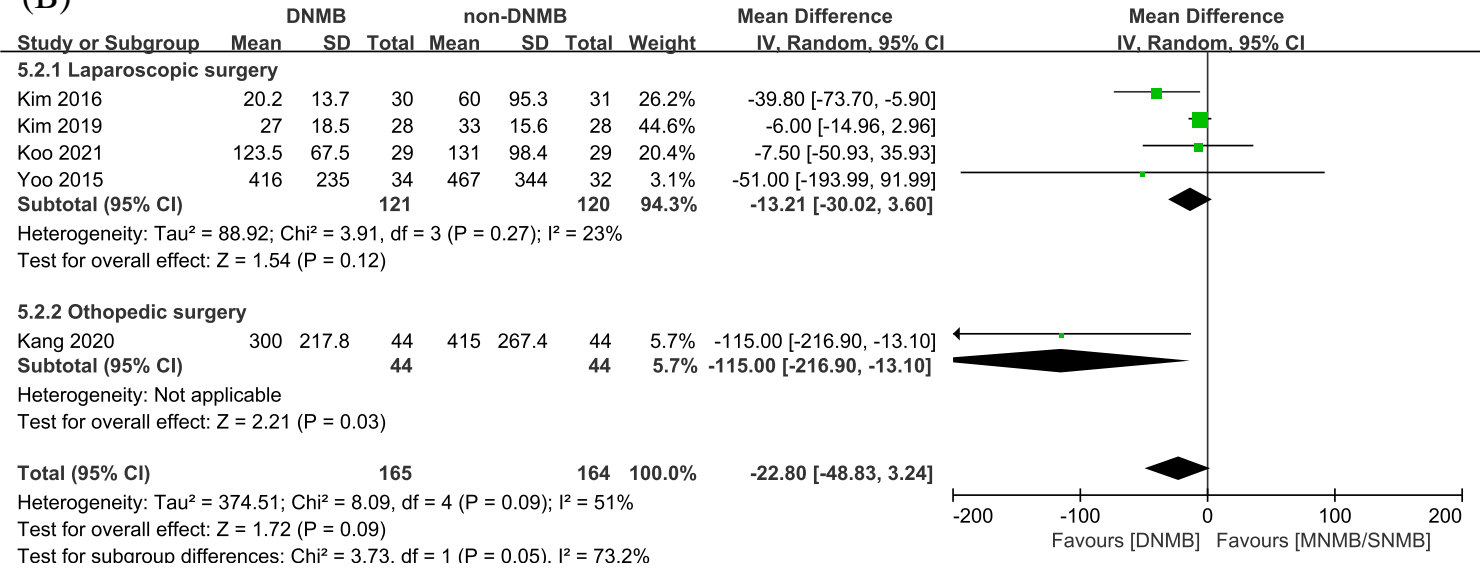

(C)

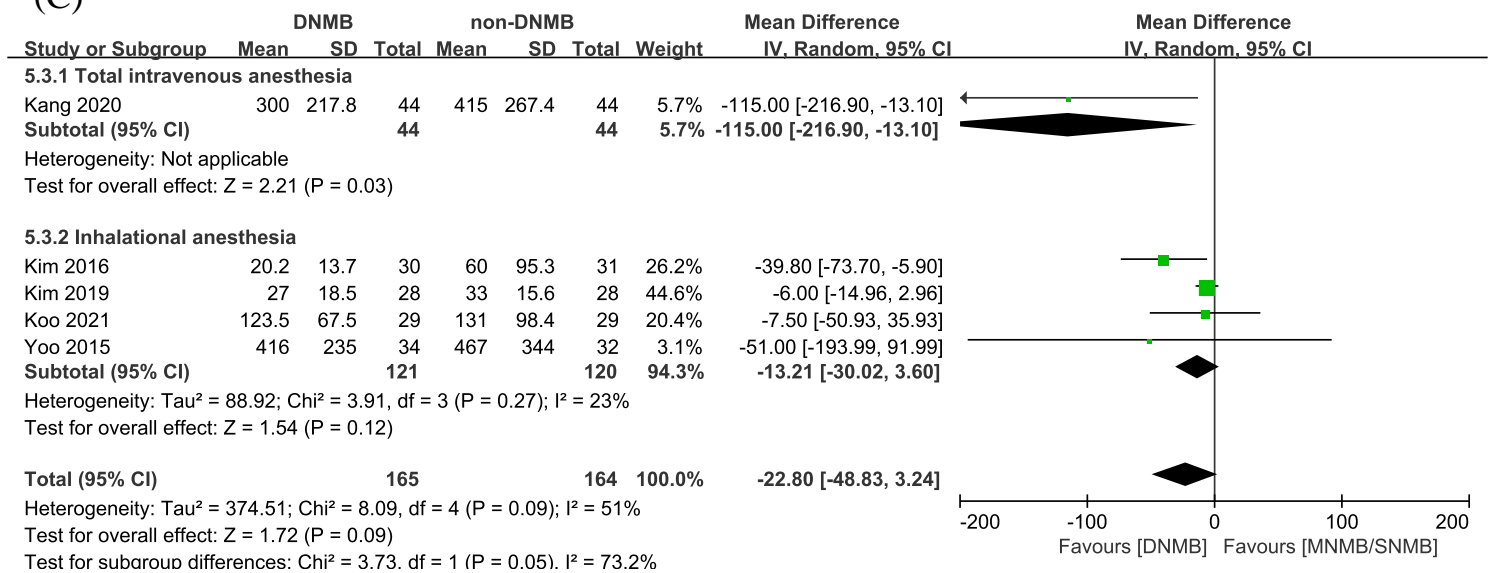

Supplement: S5 Fig — (A) The primary analysis, (B) Subgroup based on the type of surgery, (C) Subgroup based on the type of anesthesia. (PDF) [file pone.0282790.s005.pdf]

(A)

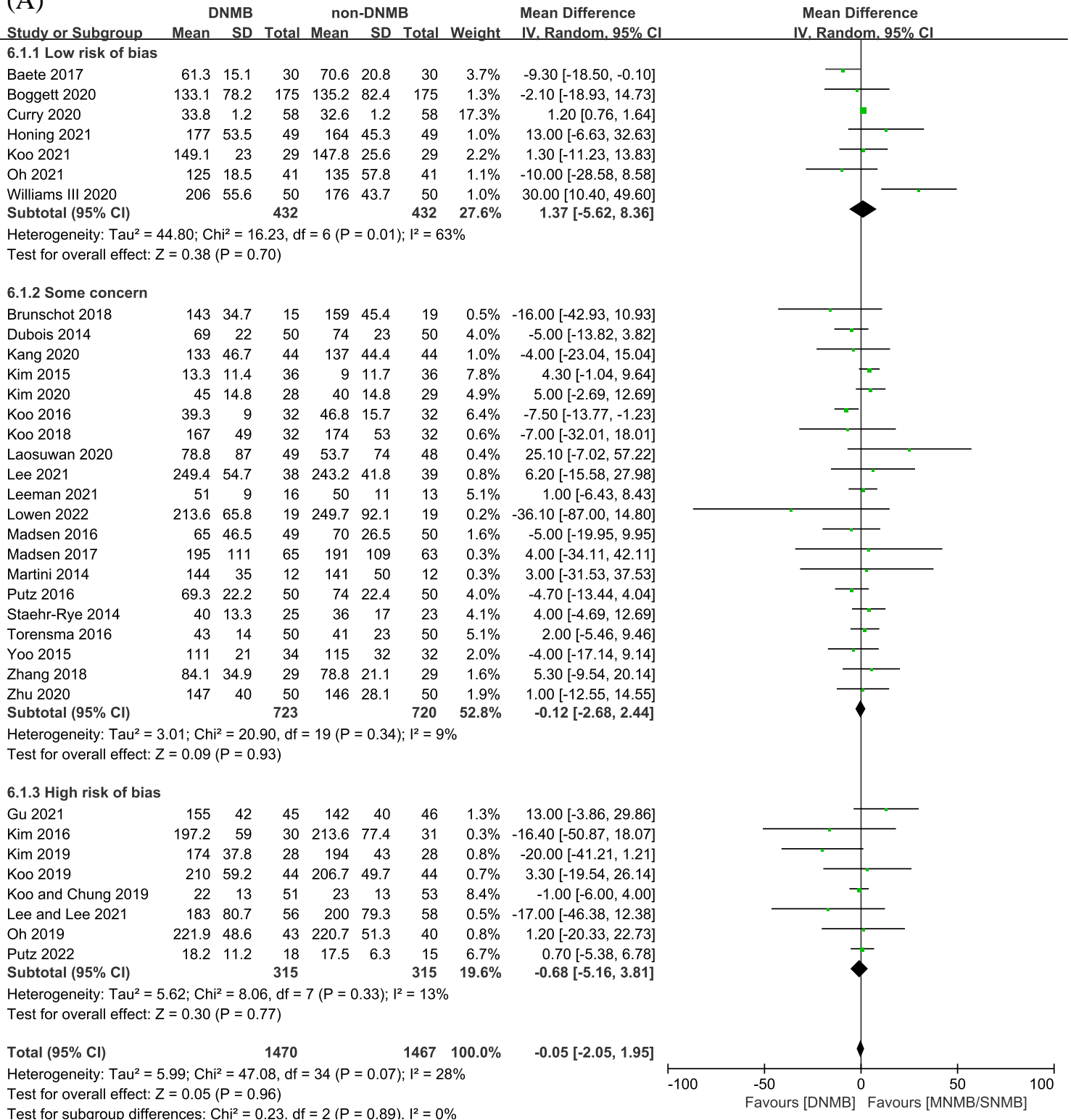

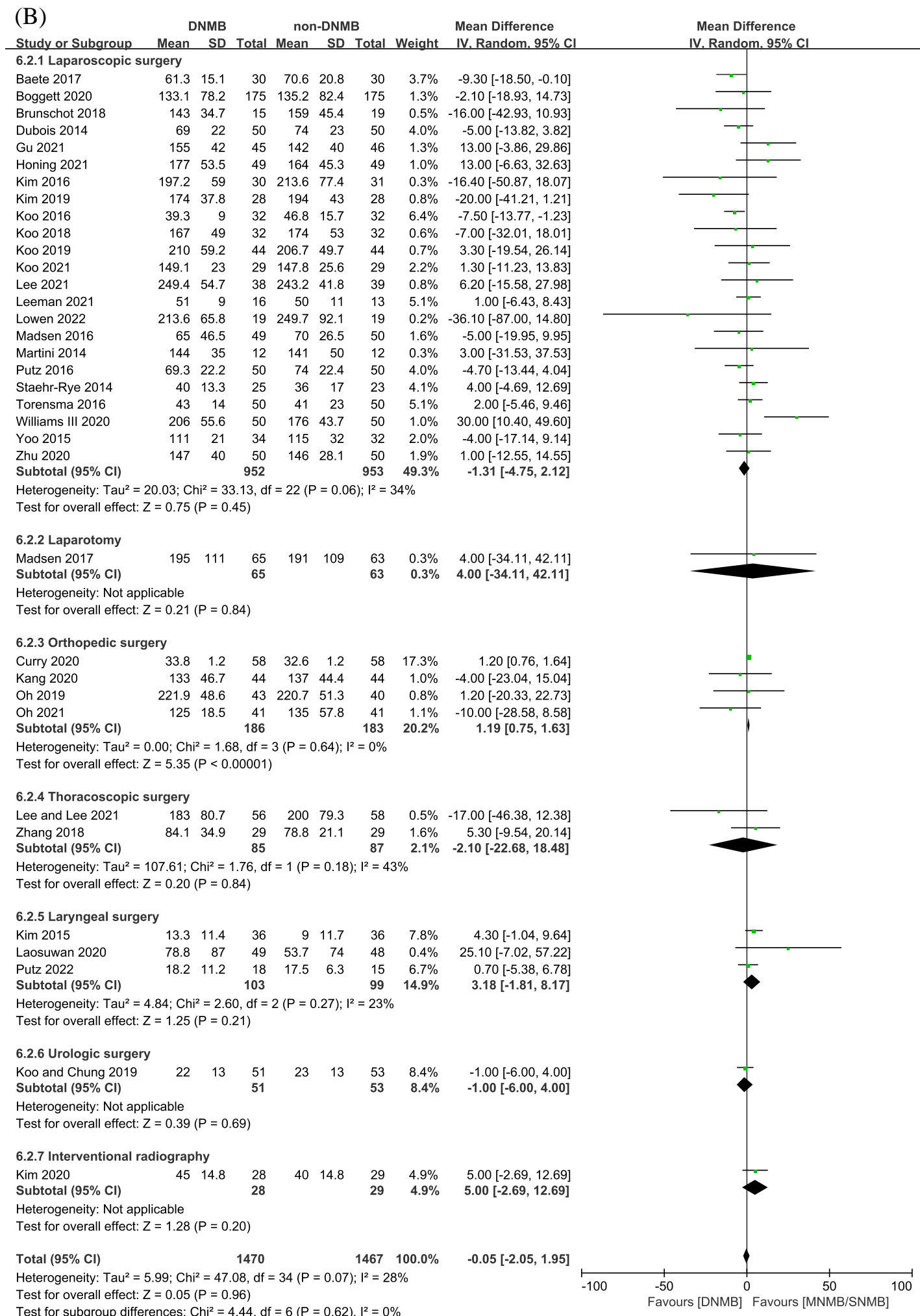

(C)

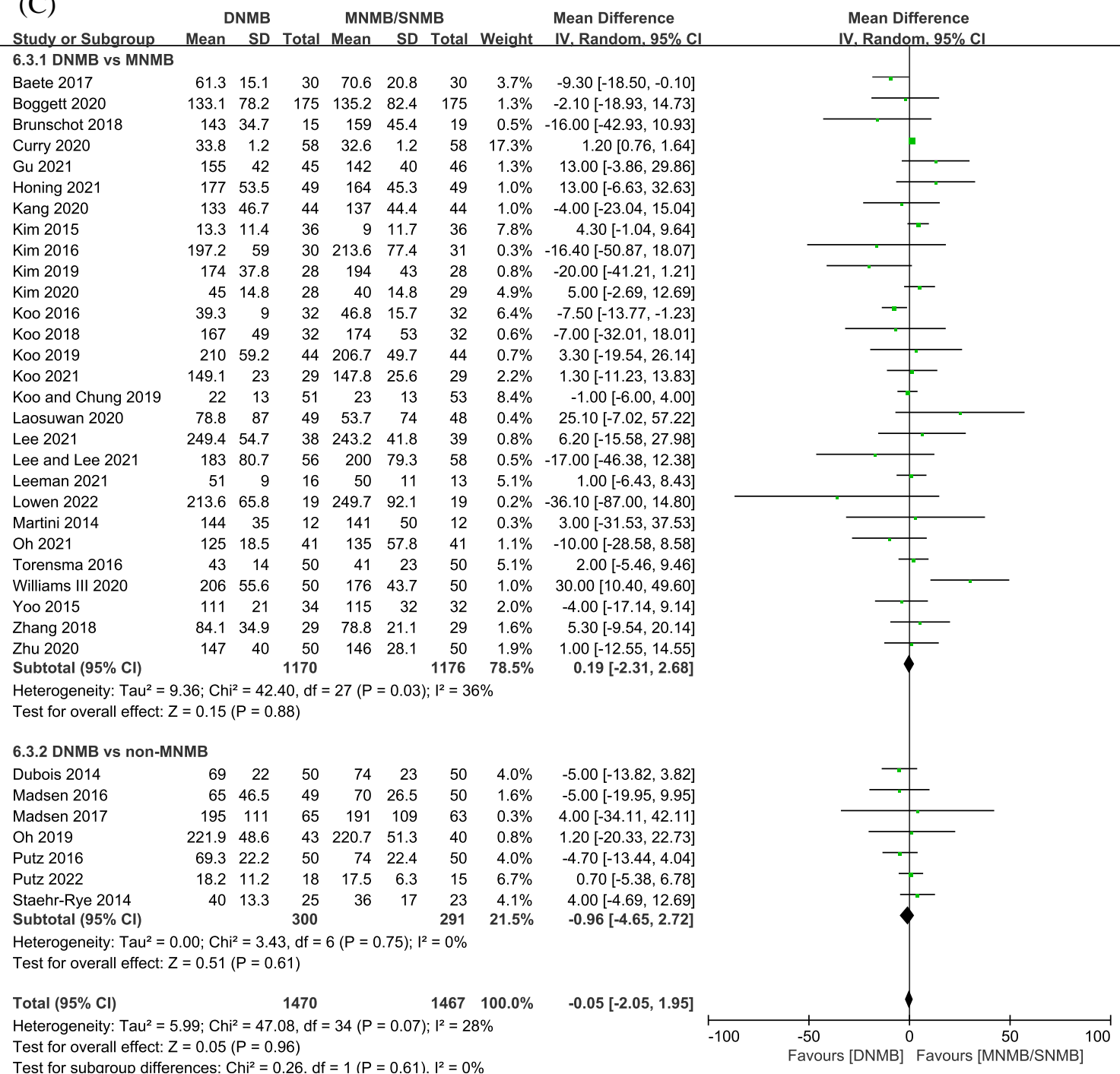

(D)

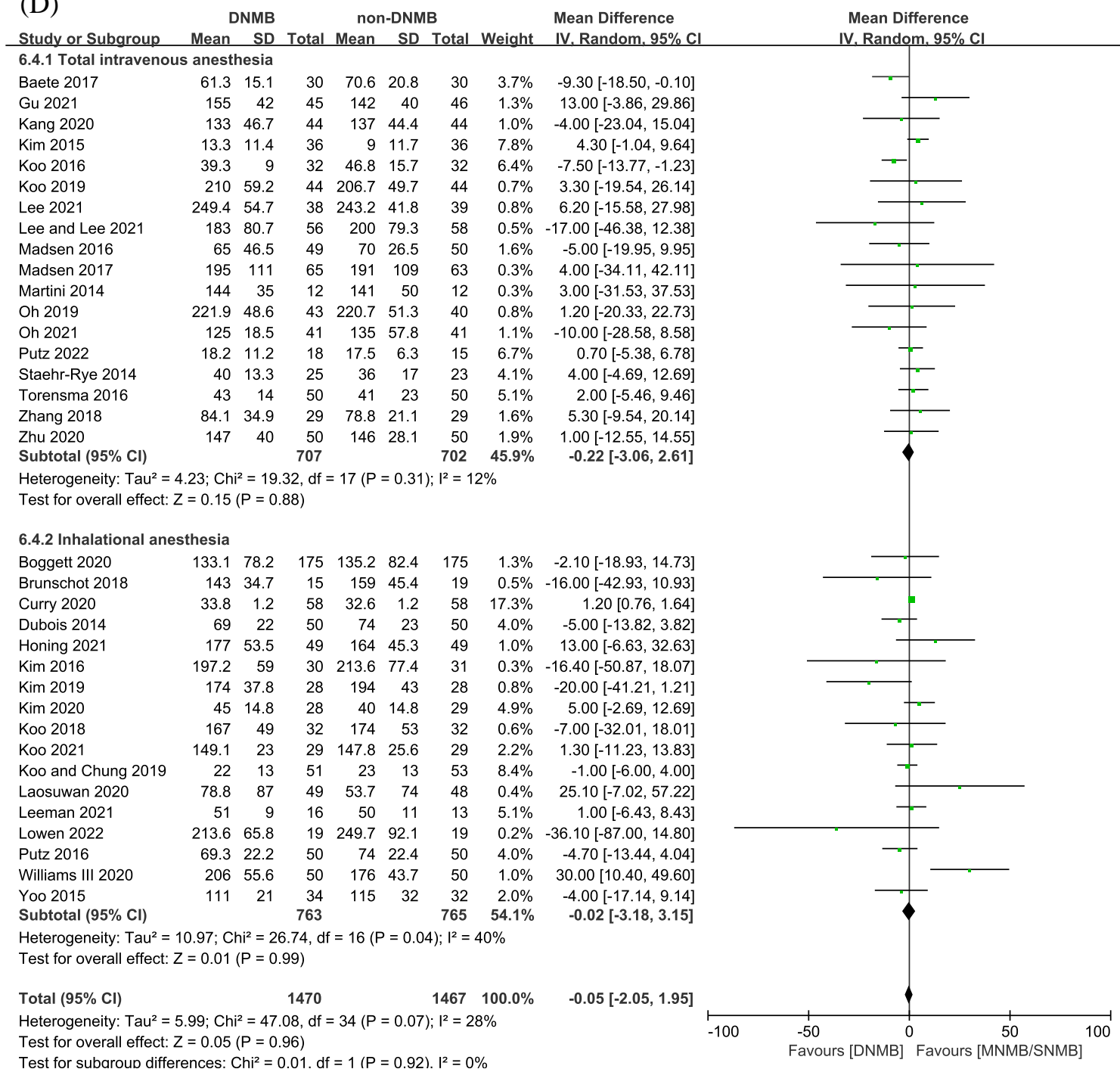

Supplement: S6 Fig — (A) Primary analysis, (B) Subgroup based on the type of surgery, (C) Subgroup based on the depth of neuromuscular blockade, (D) Subgroup based on the type of anesthesia. (PDF) [file pone.0282790.s006.pdf]

(A)

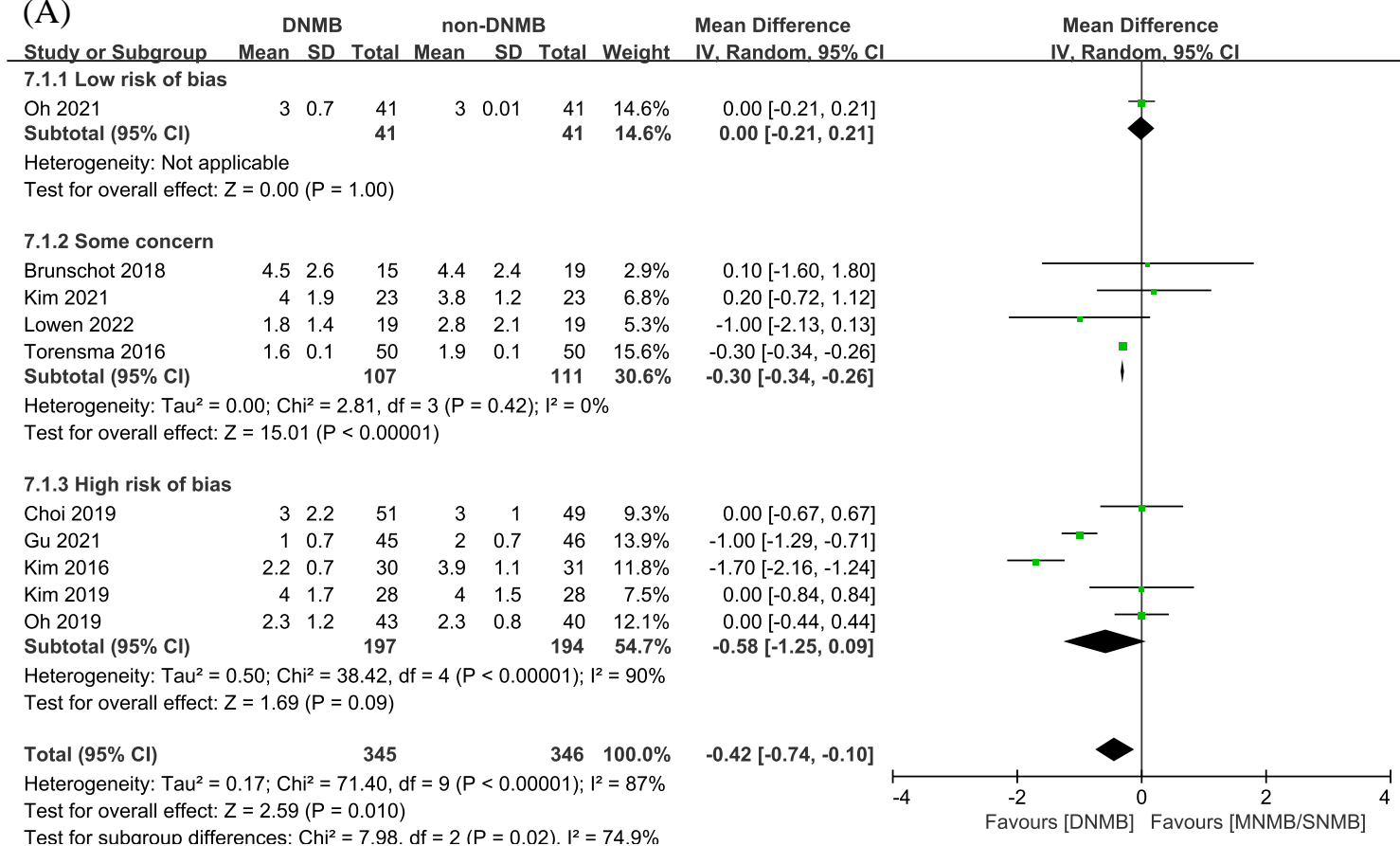

(B)

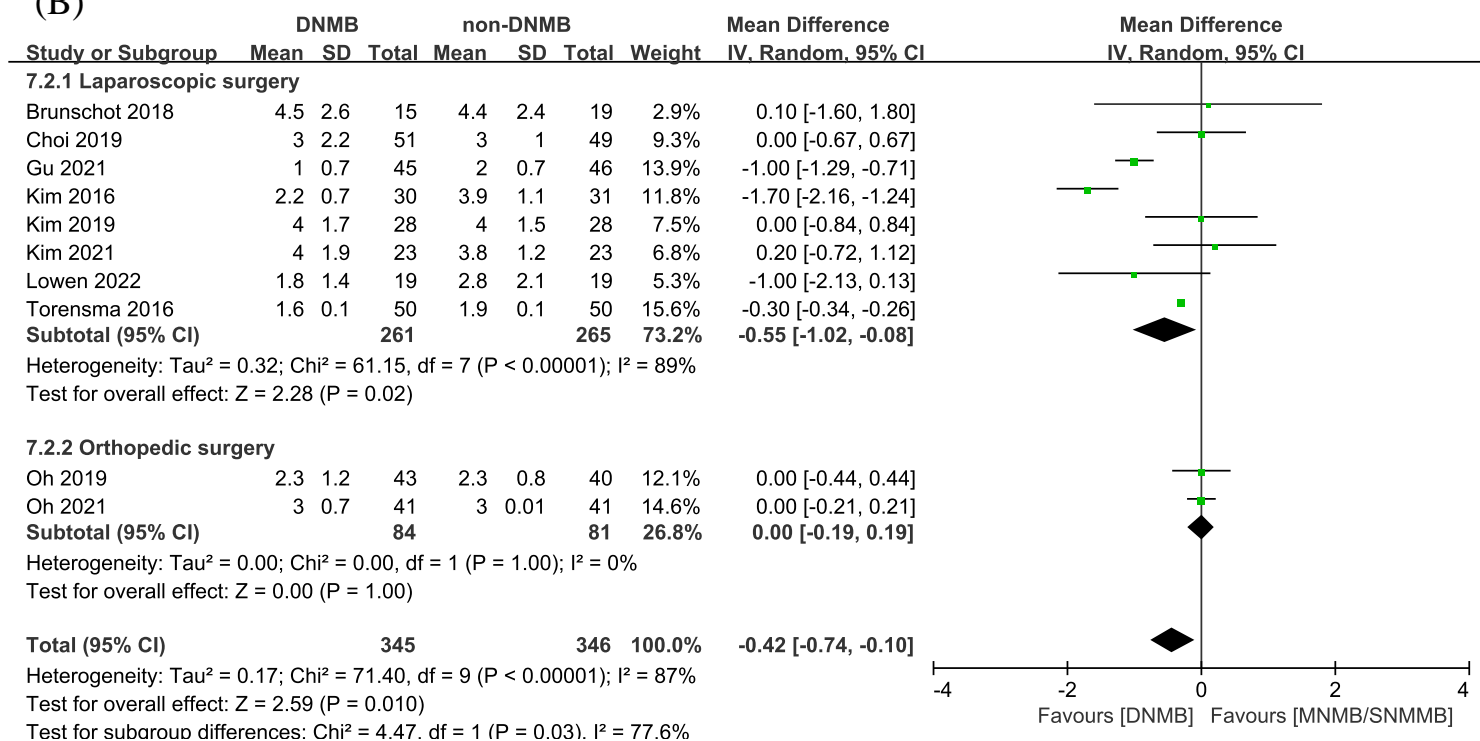

(C)

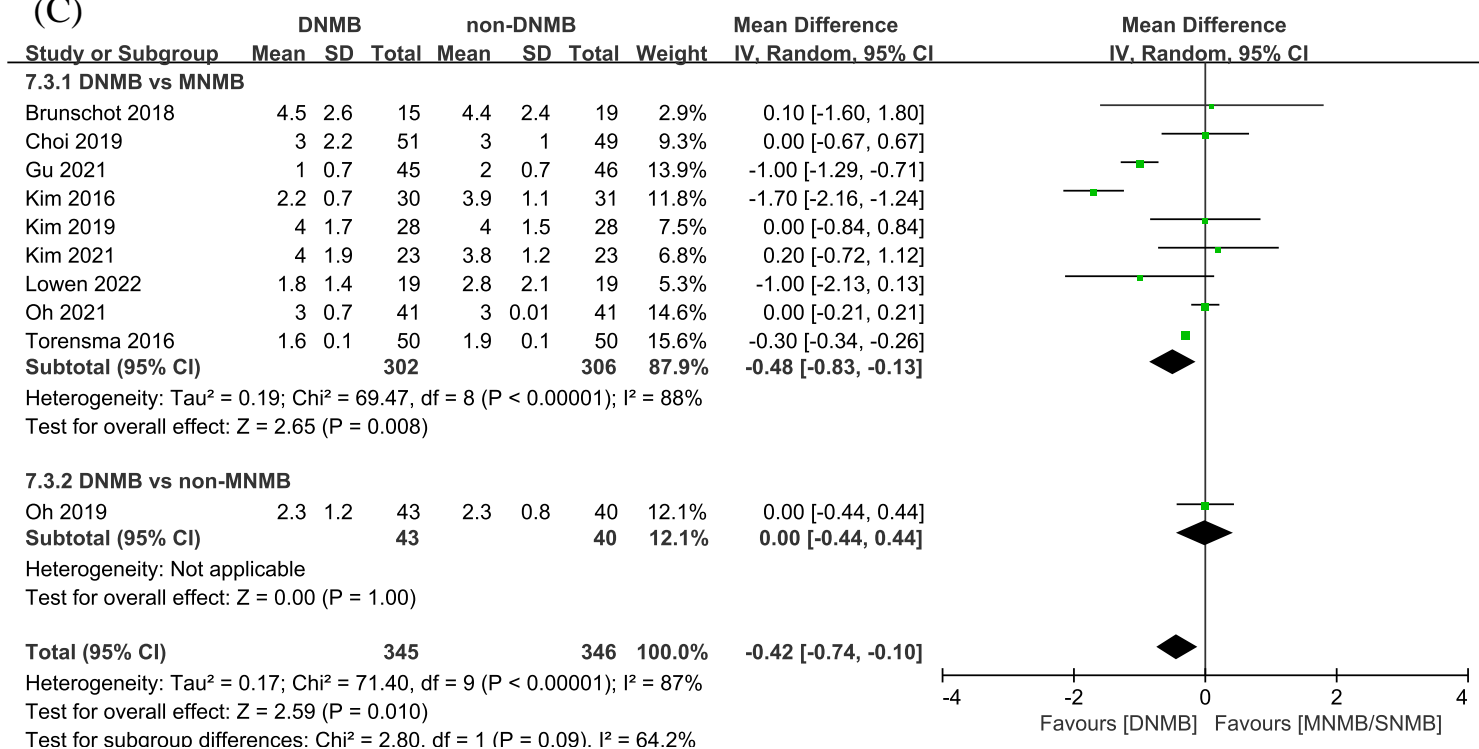

(D)

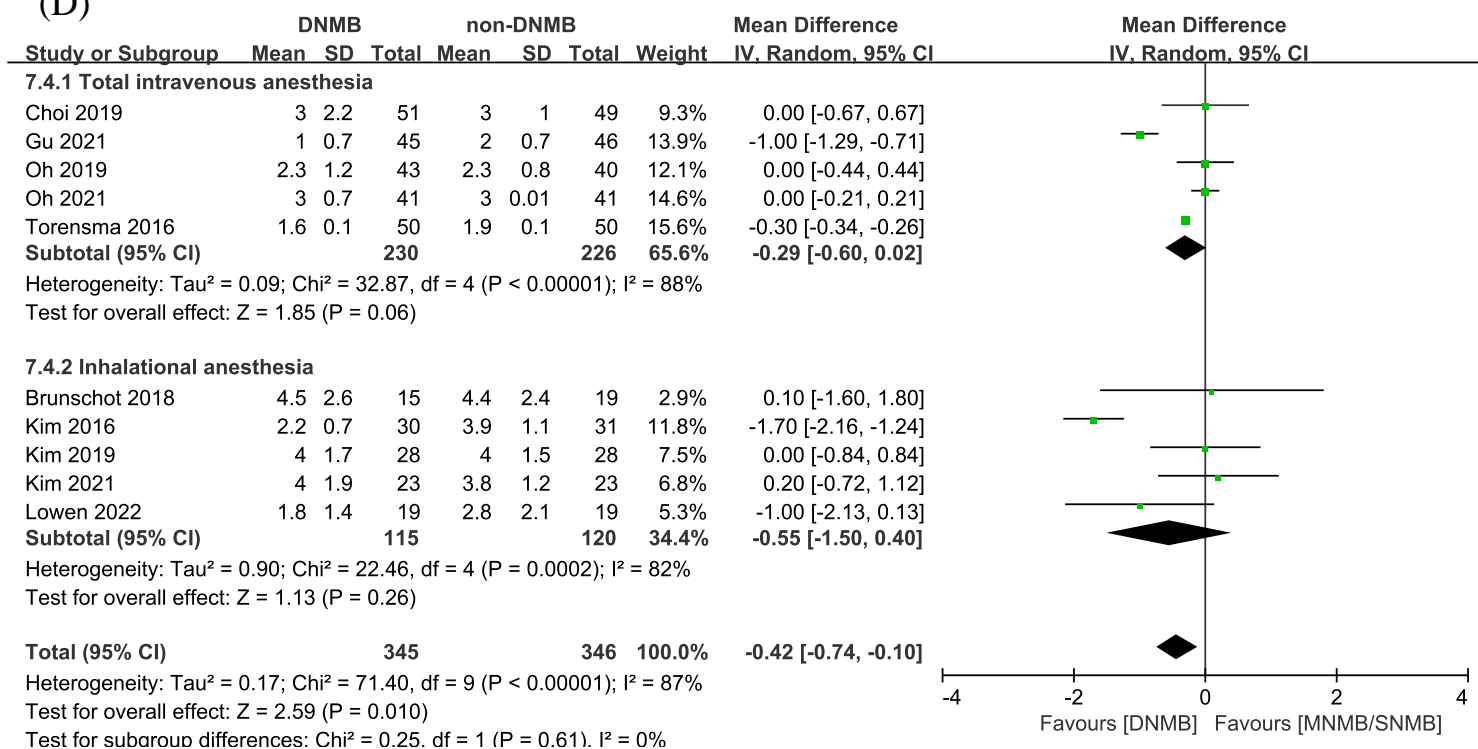

Supplement: S7 Fig — (A) Primary analysis, (B) Subgroup based on the type of surgery, (C) Subgroup based on the depth of neuromuscular blockade, (D) Subgroup based on the type of anesthesia. (PDF) [file pone.0282790.s007.pdf]

(A)

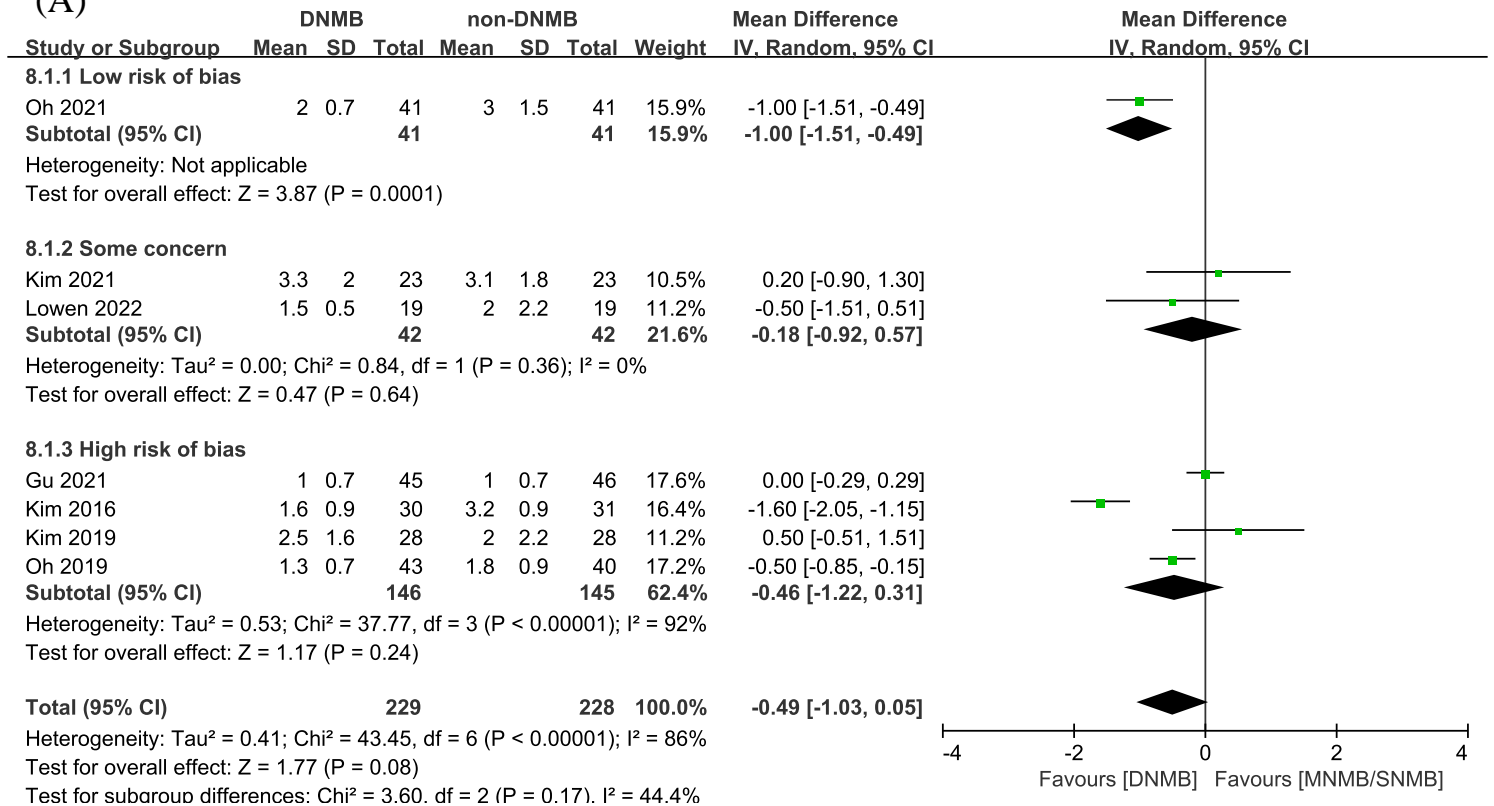

(B)

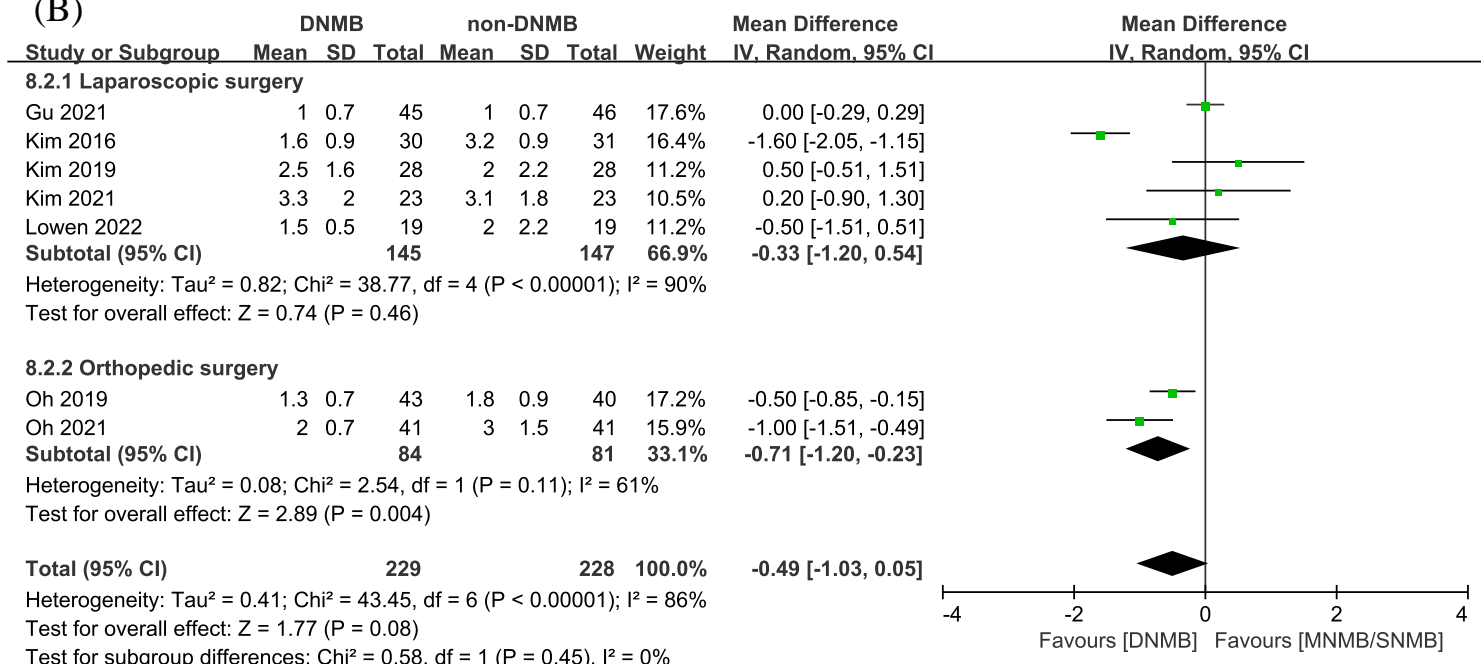

(C)

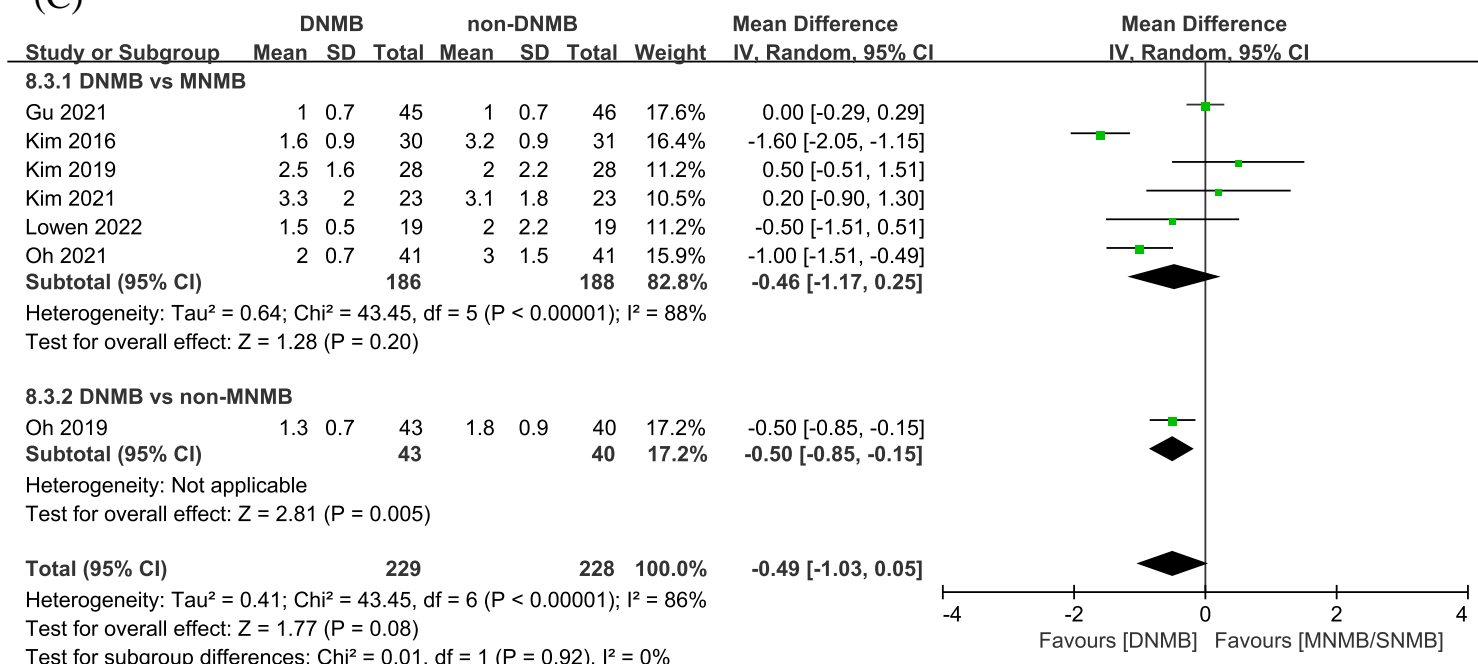

(D)

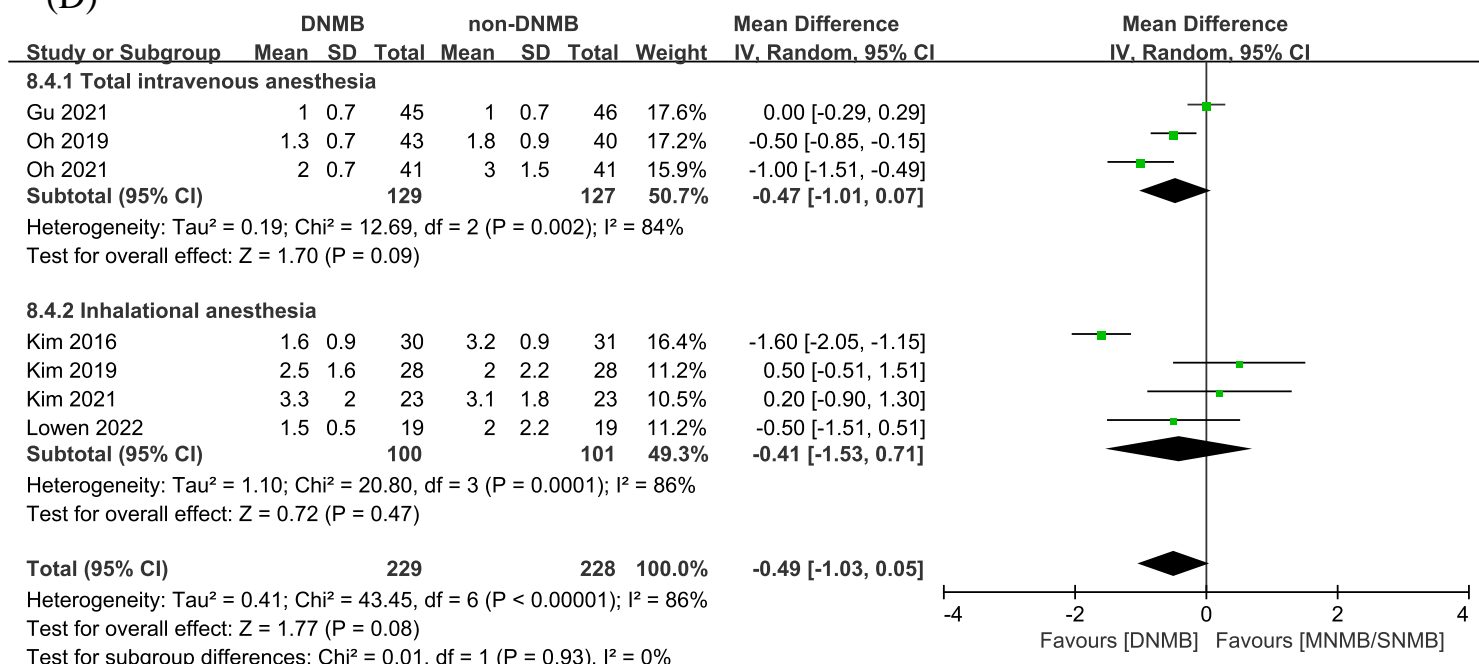

Supplement: S8 Fig — (A) Primary analysis, (B) Subgroup based on the type of surgery, (C) Subgroup based on the depth of neuromuscular blockade, (D) Subgroup based on the type of anesthesia. (PDF) [file pone.0282790.s008.pdf]

(A)

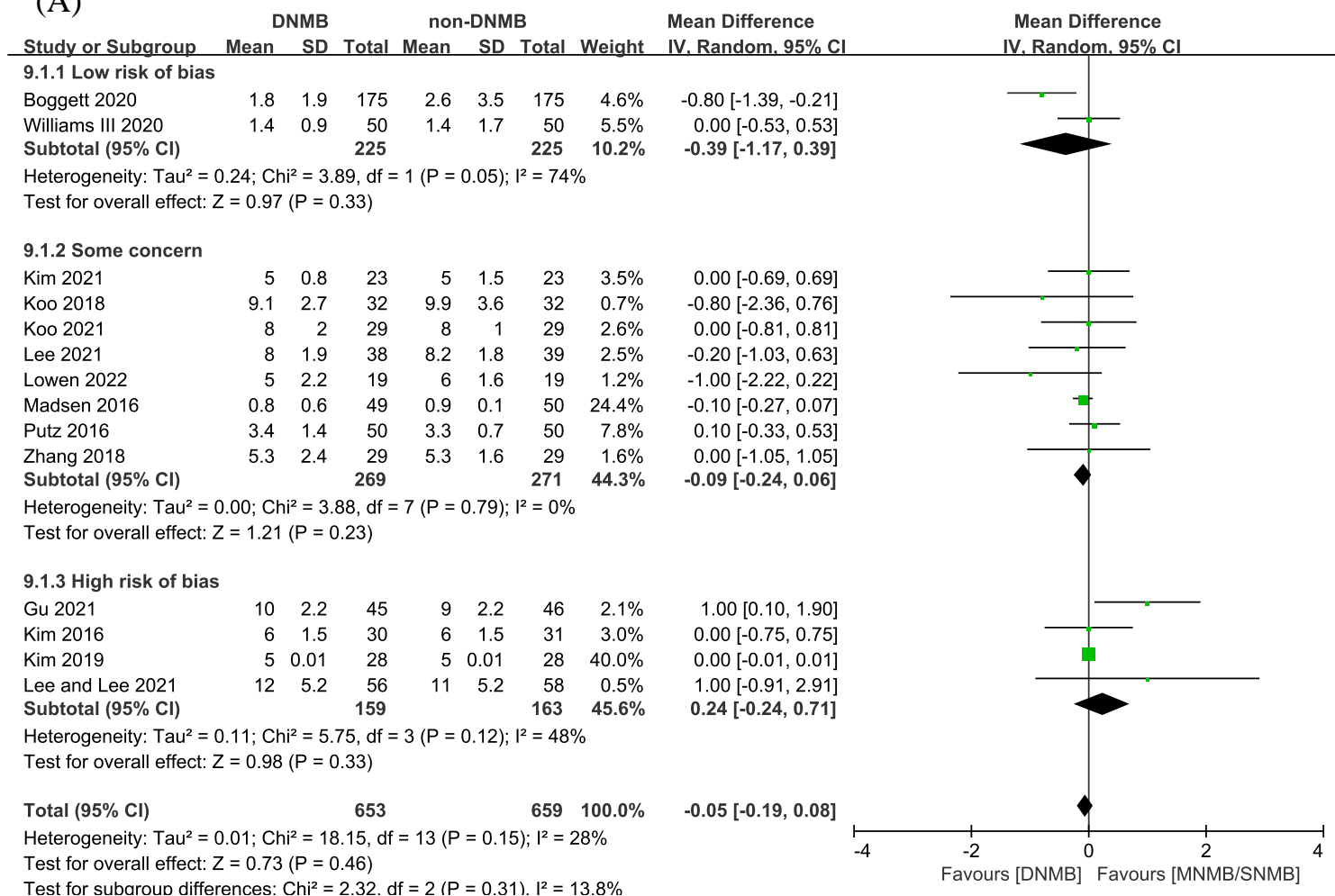

(B)

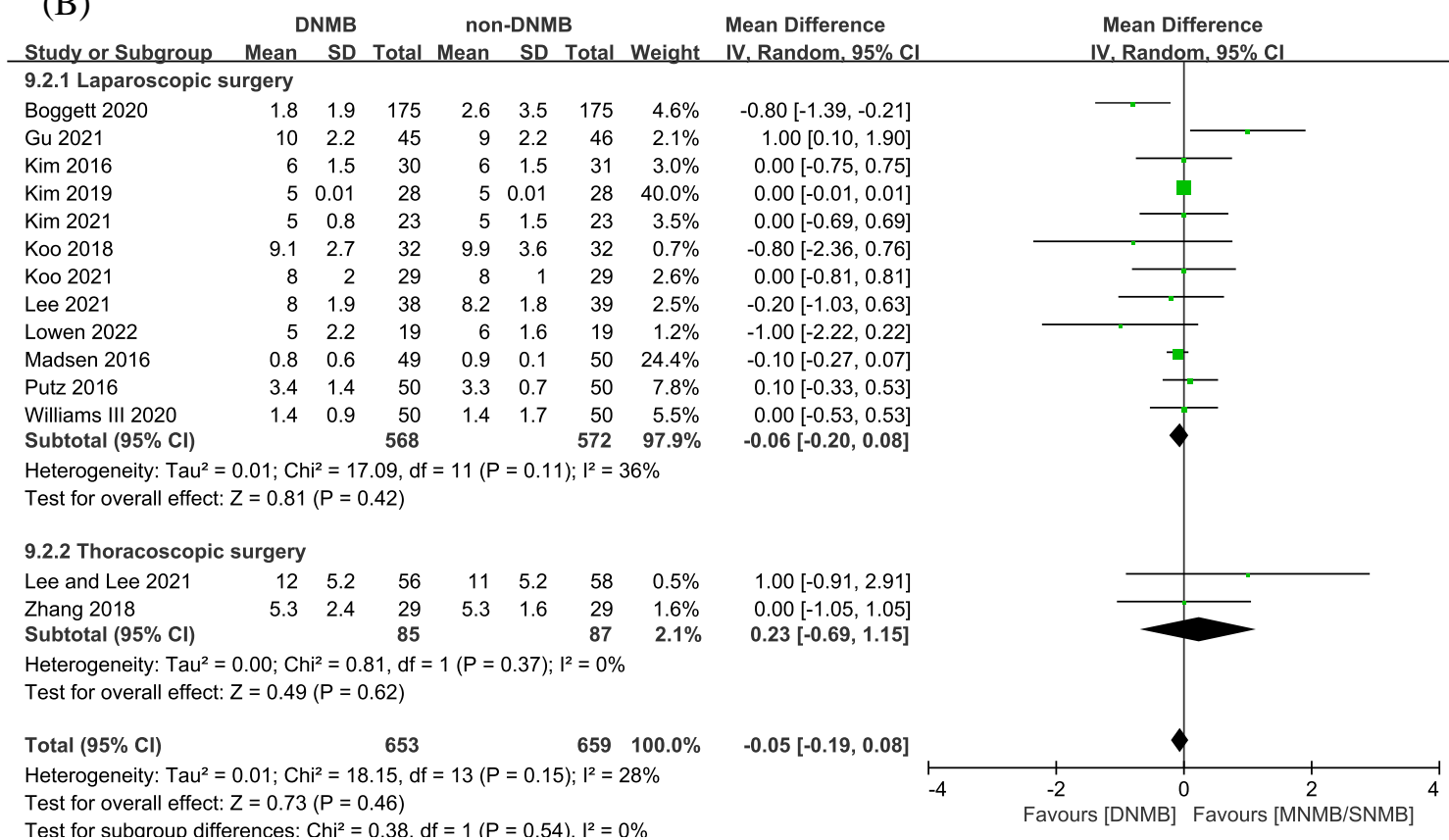

(C)

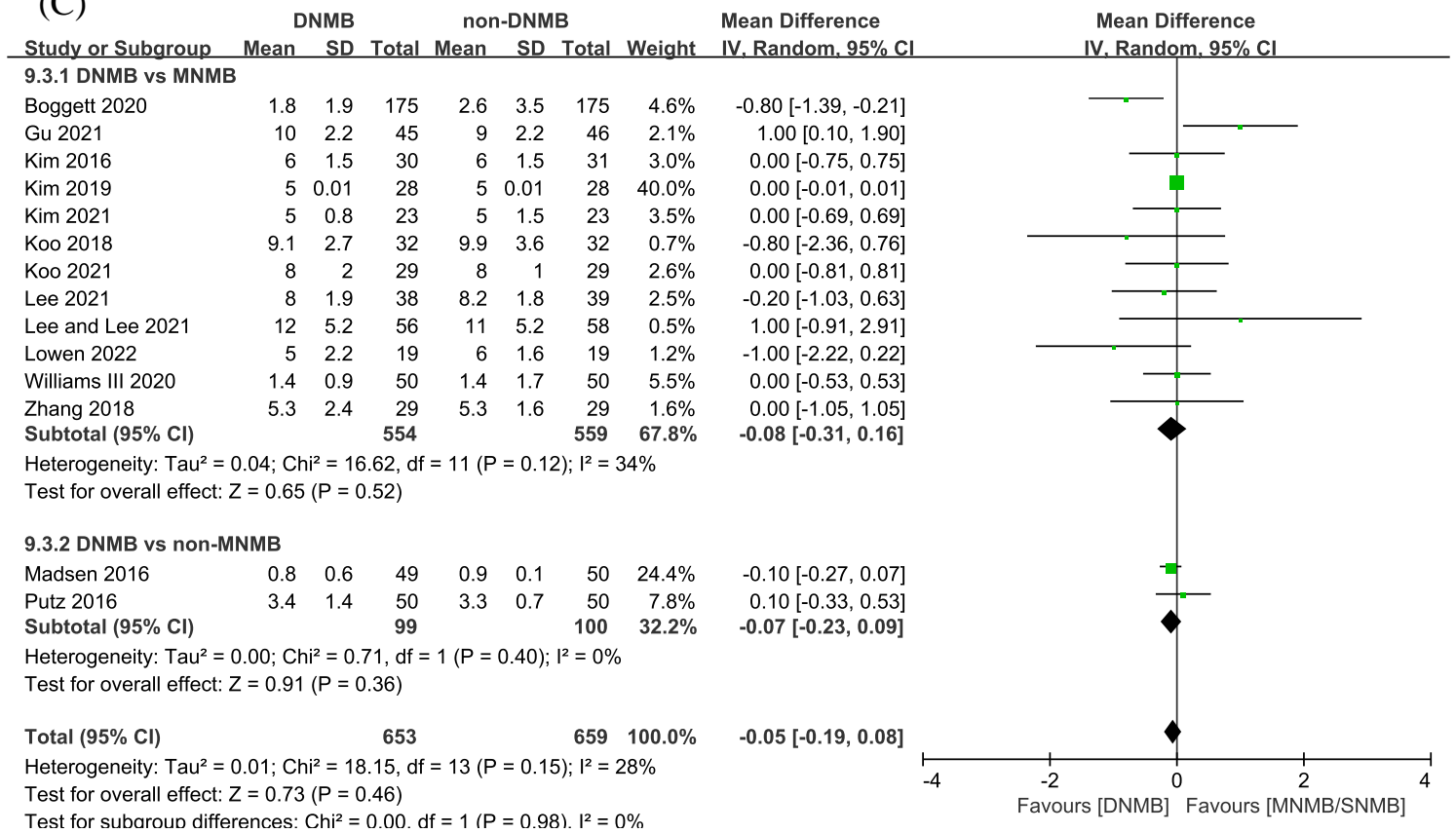

(D)

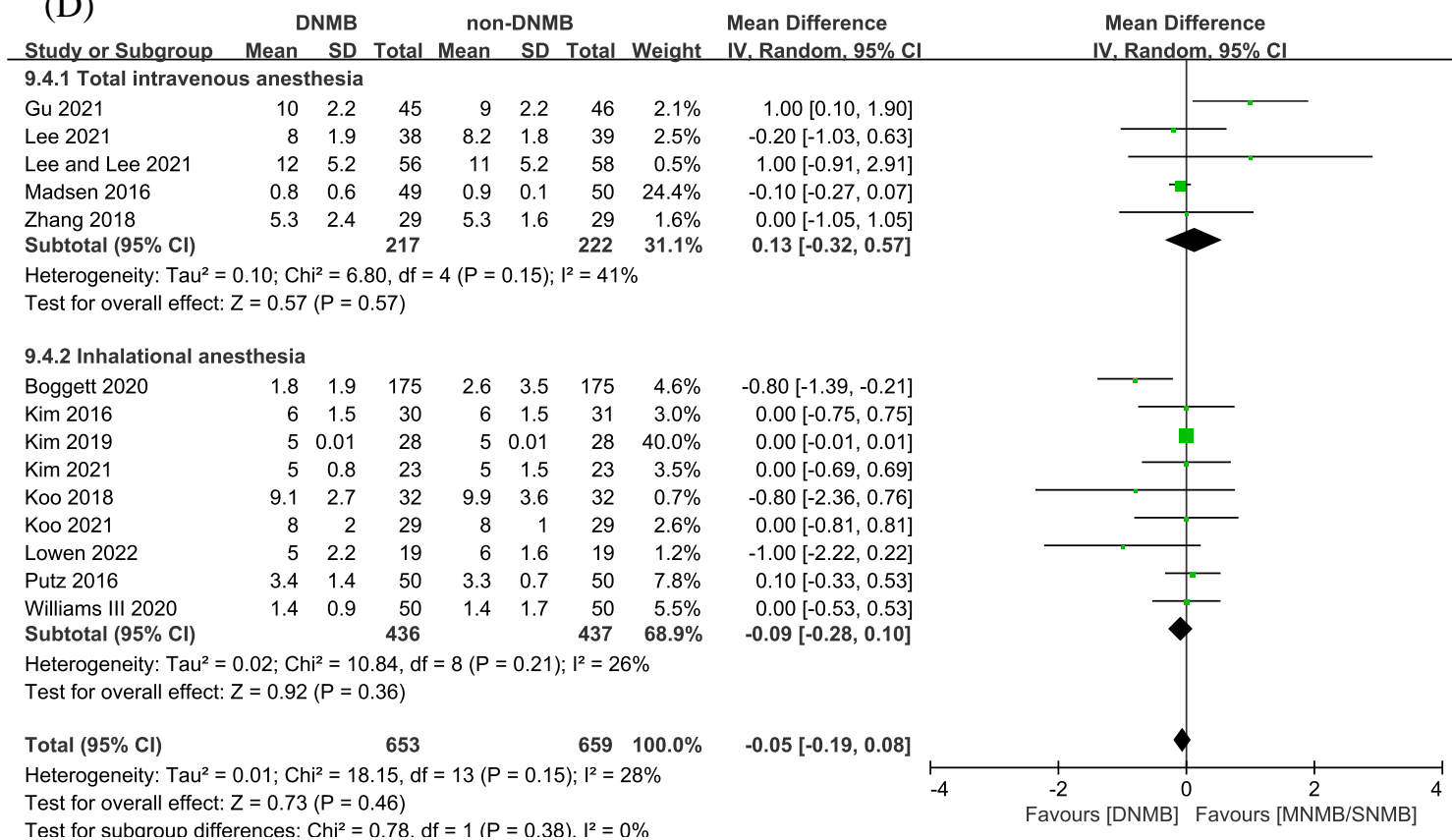

Supplement: S9 Fig — (A) Primary analysis, (B) Subgroup based on the type of surgery, (C) Subgroup based on the depth of neuromuscular blockade, (D) Subgroup based on the type of anesthesia. (PDF) [file pone.0282790.s009.pdf]

(A)

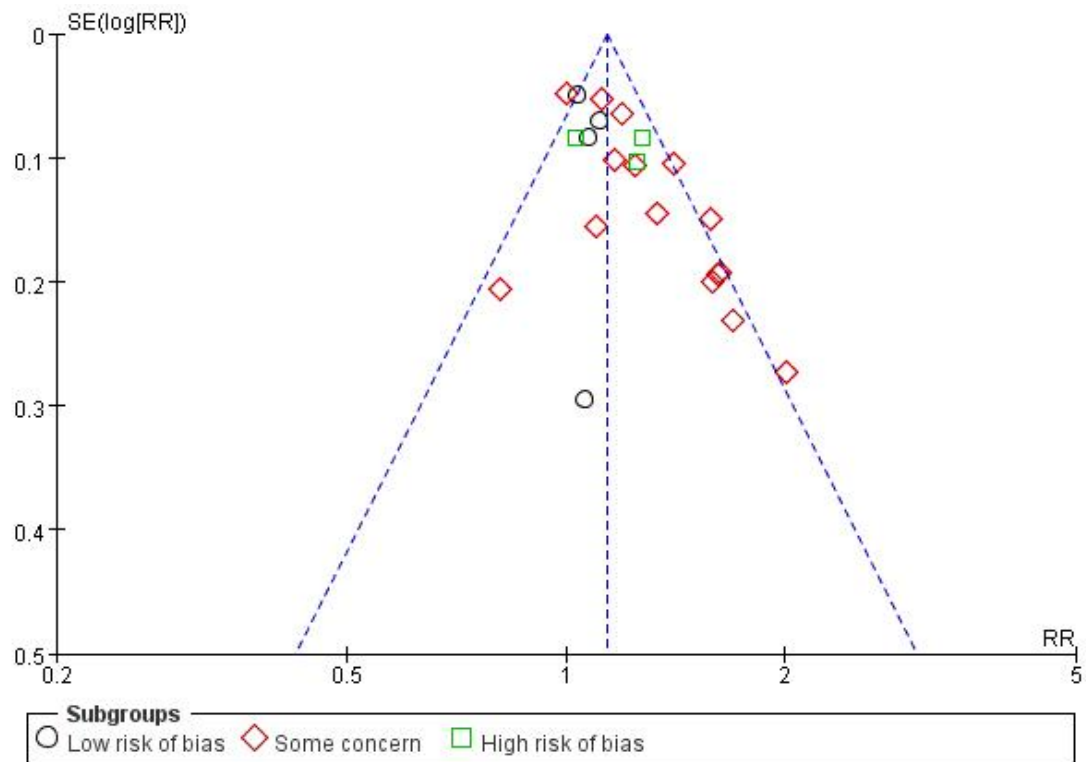

(B)

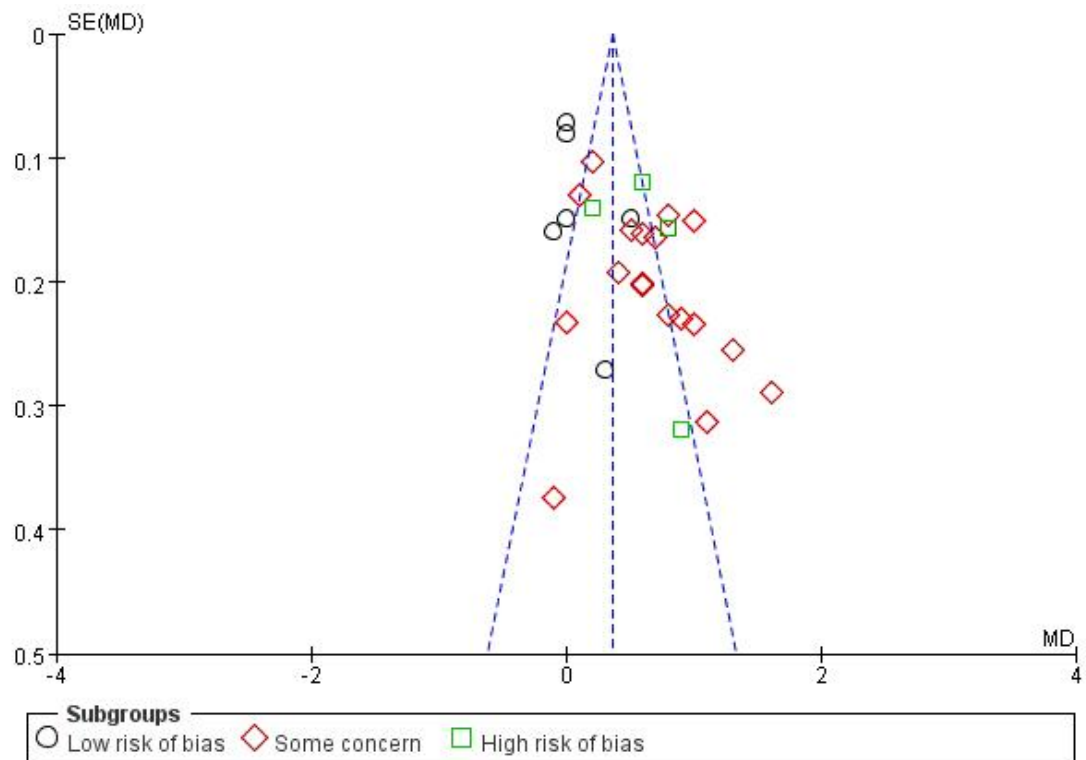

(C)

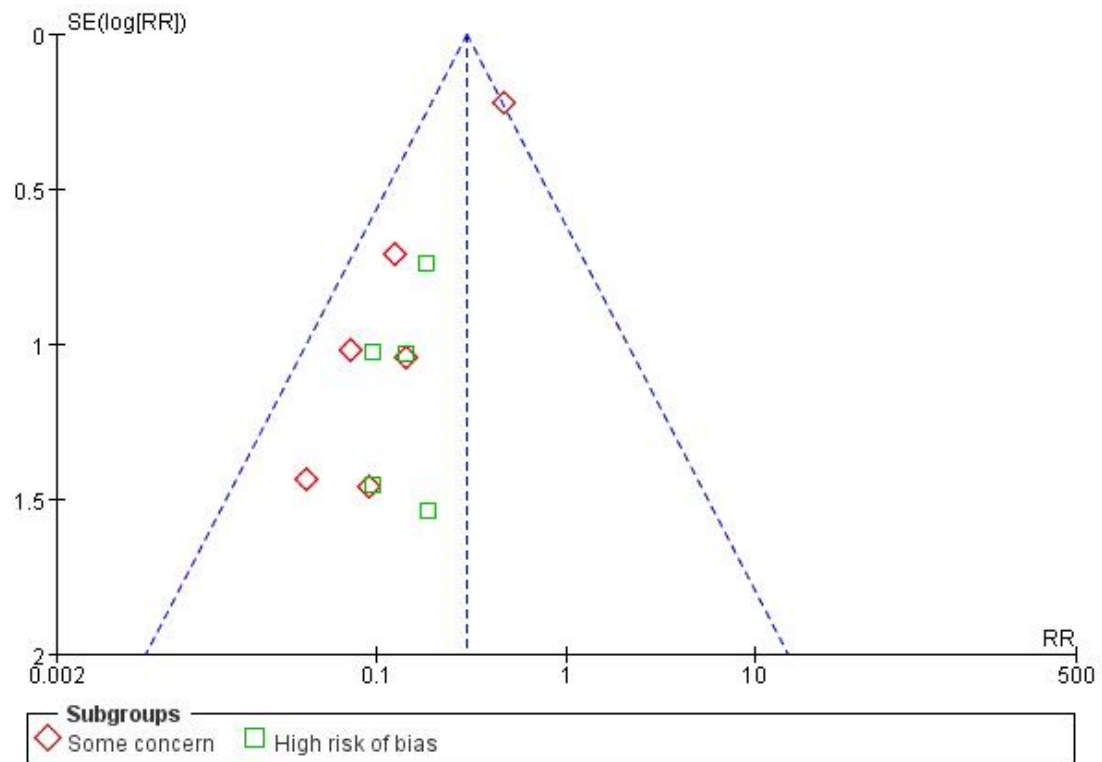

(D)

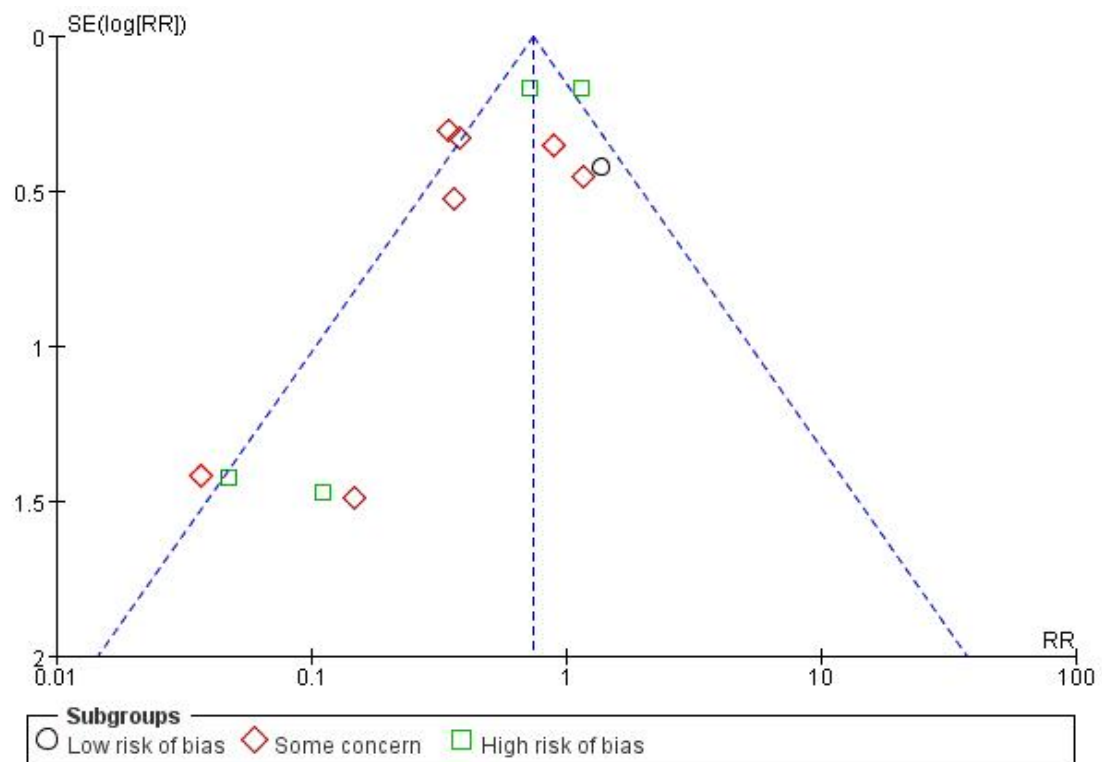

(E)

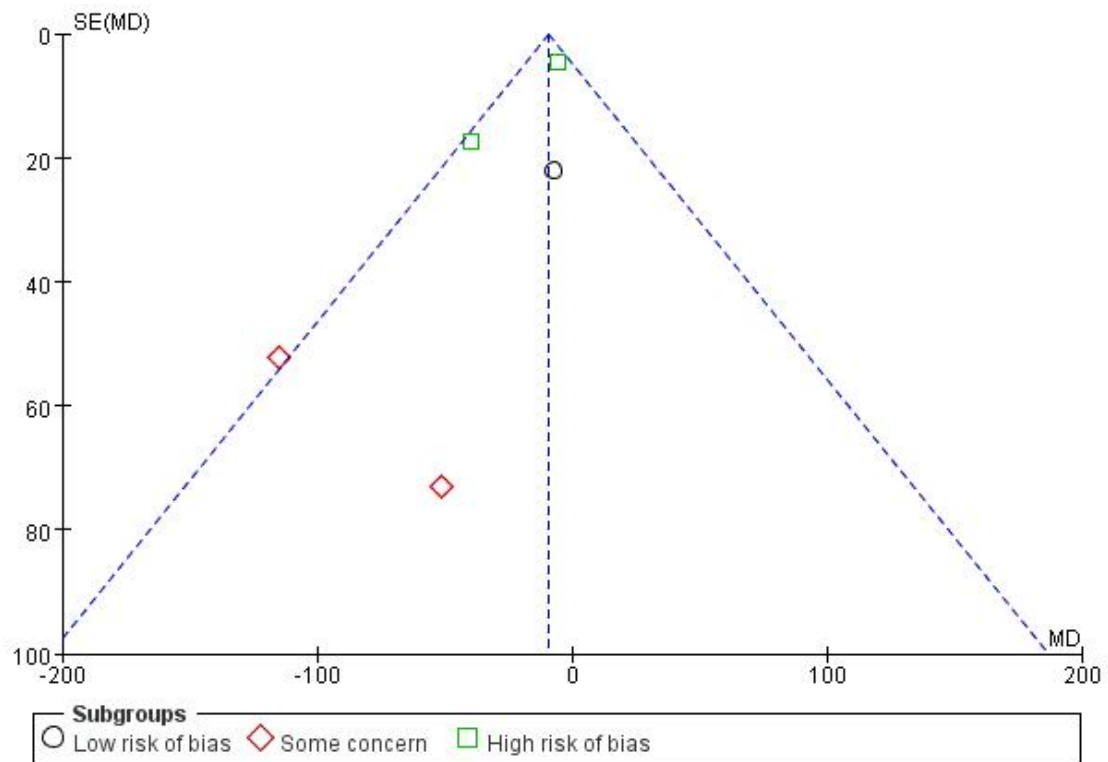

(F)

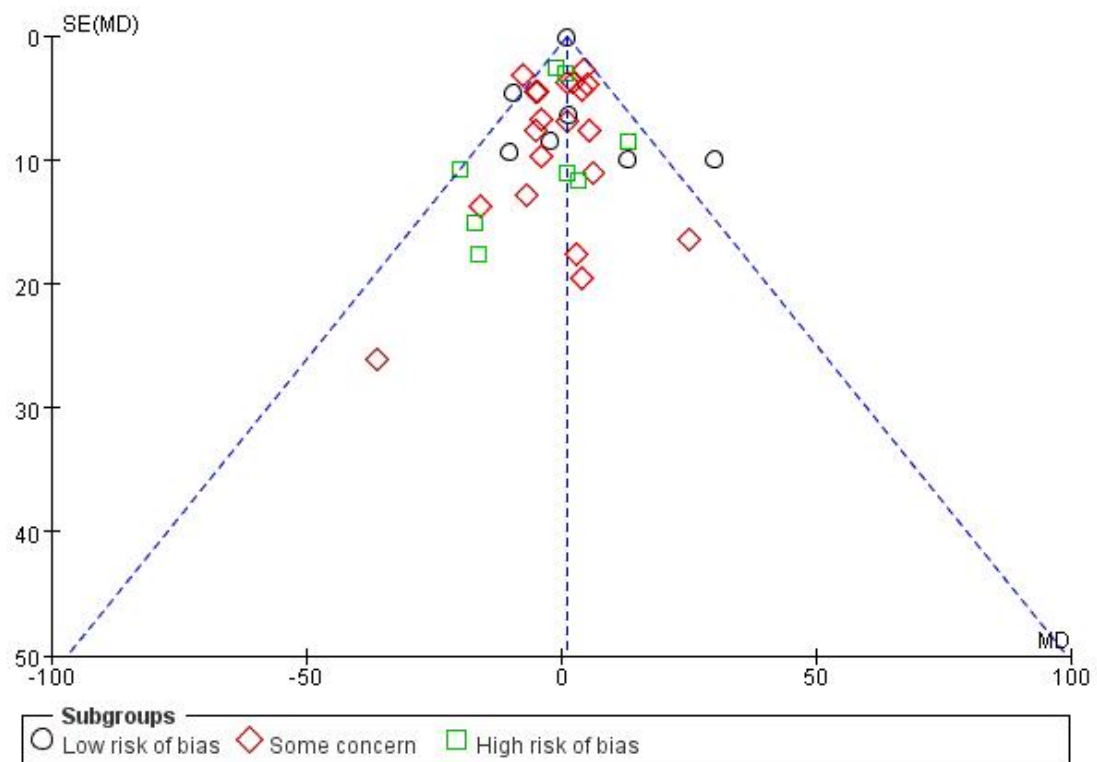

(G)

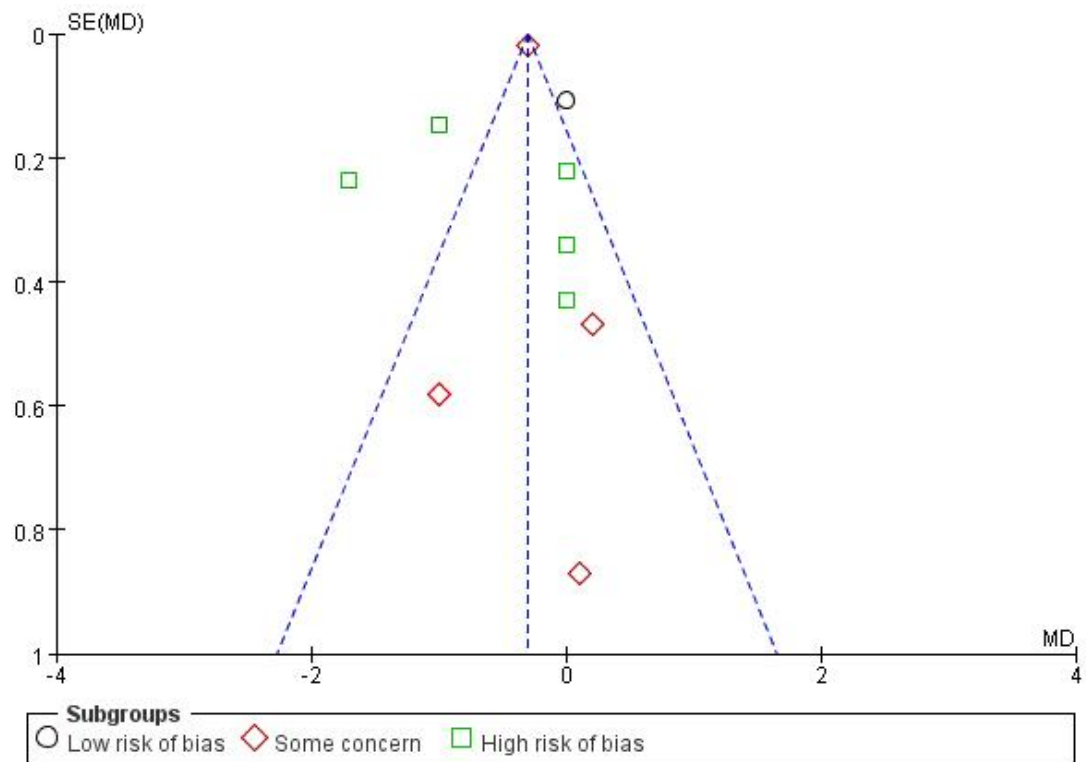

(H)

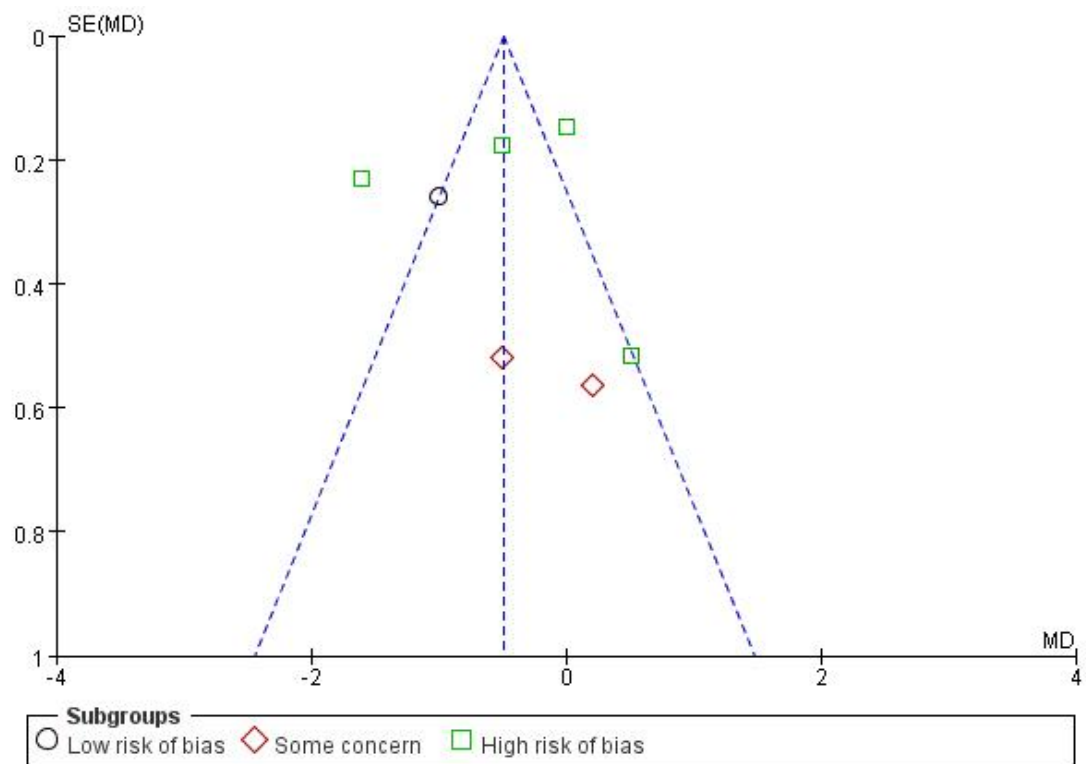

(I)

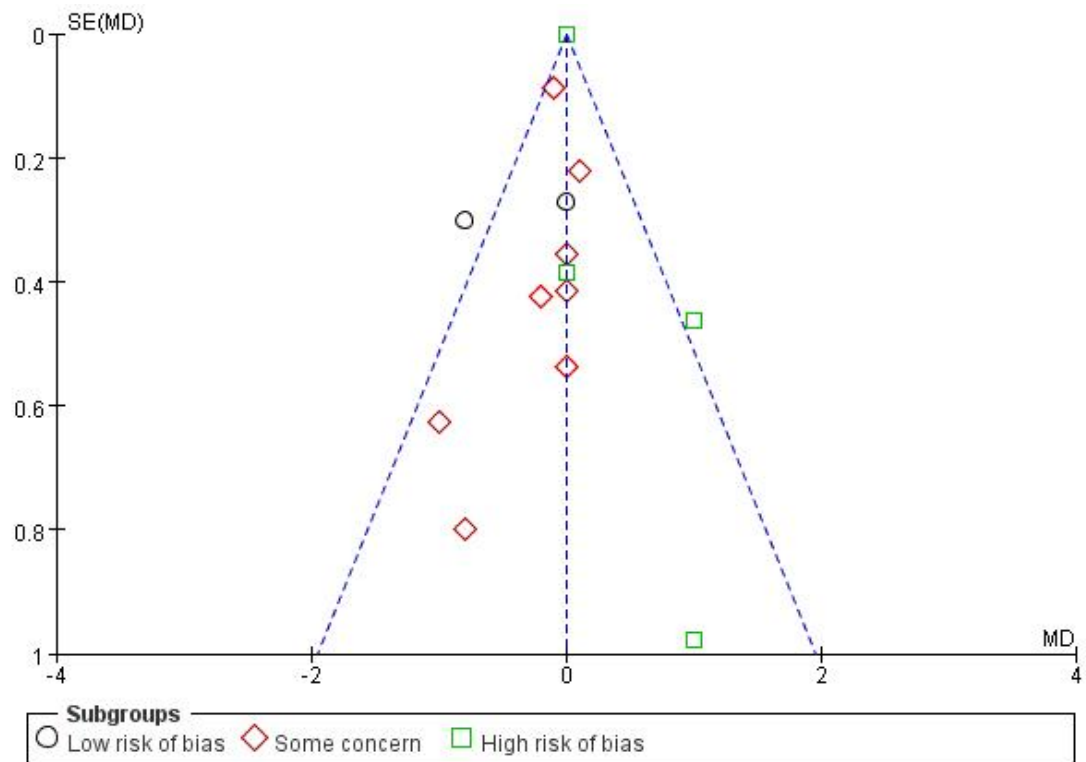

Supplement: S10 Fig — (A) Acceptable surgical condition, (B) Surgical condition score, (C) Intraoperative movement, (D) Additional measure to improve the surgical condition, (E) Intraoperative blood loss, (F) Duration of surgery, (G) Pain at 24 h, (H) Pain at 48 h, (I) Length of stay. (PDF) [file pone.0282790.s010.pdf]

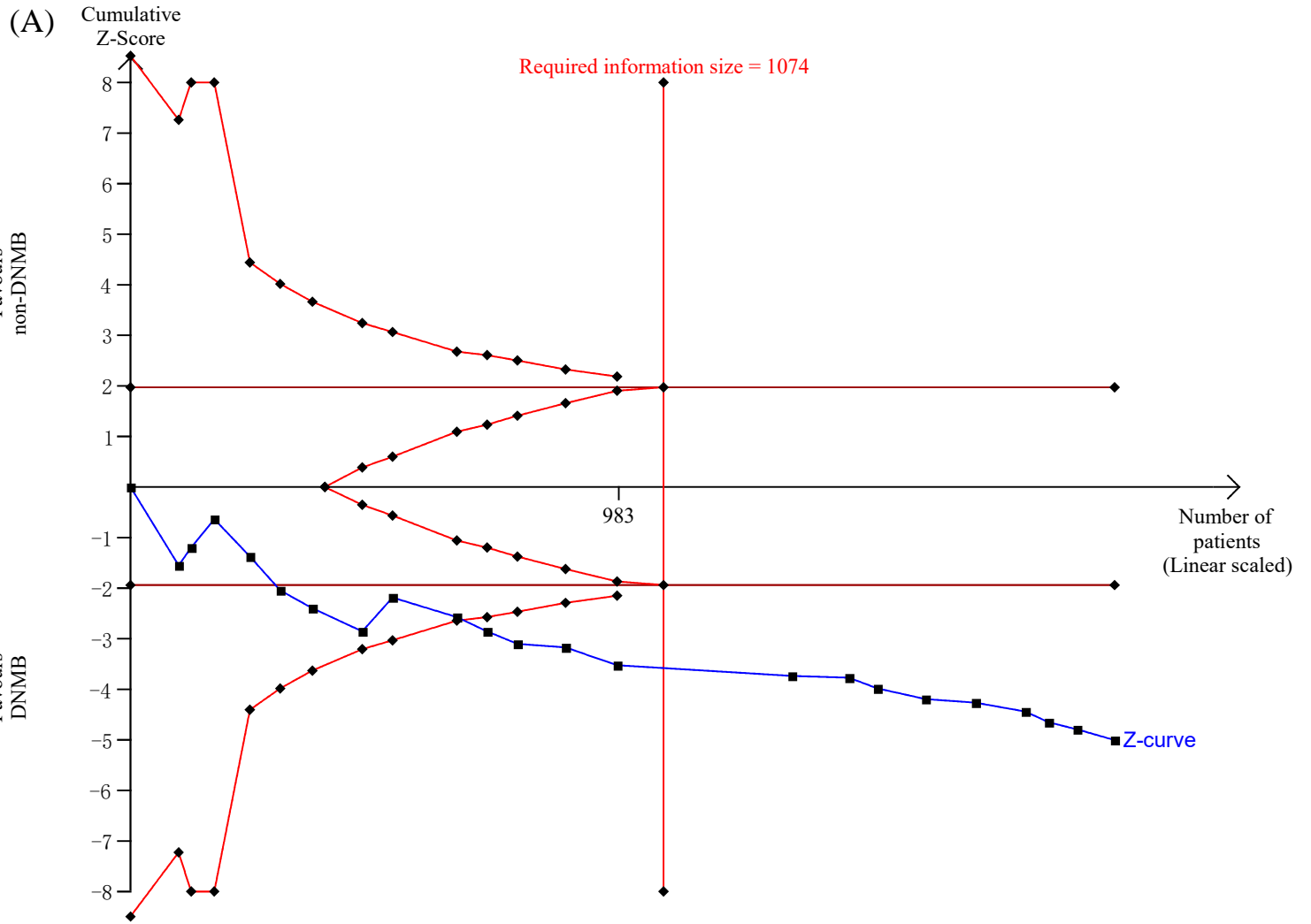

(B)

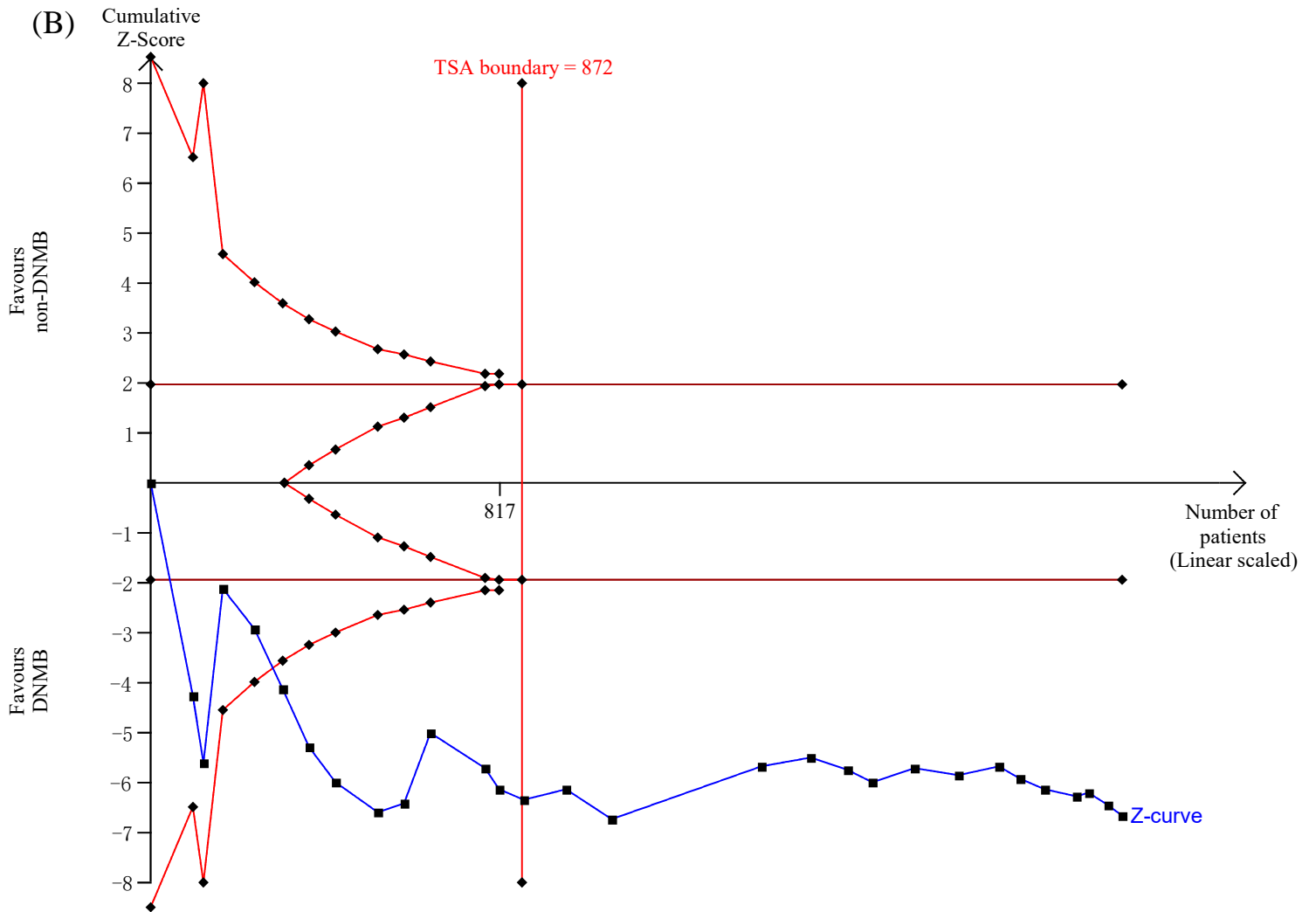

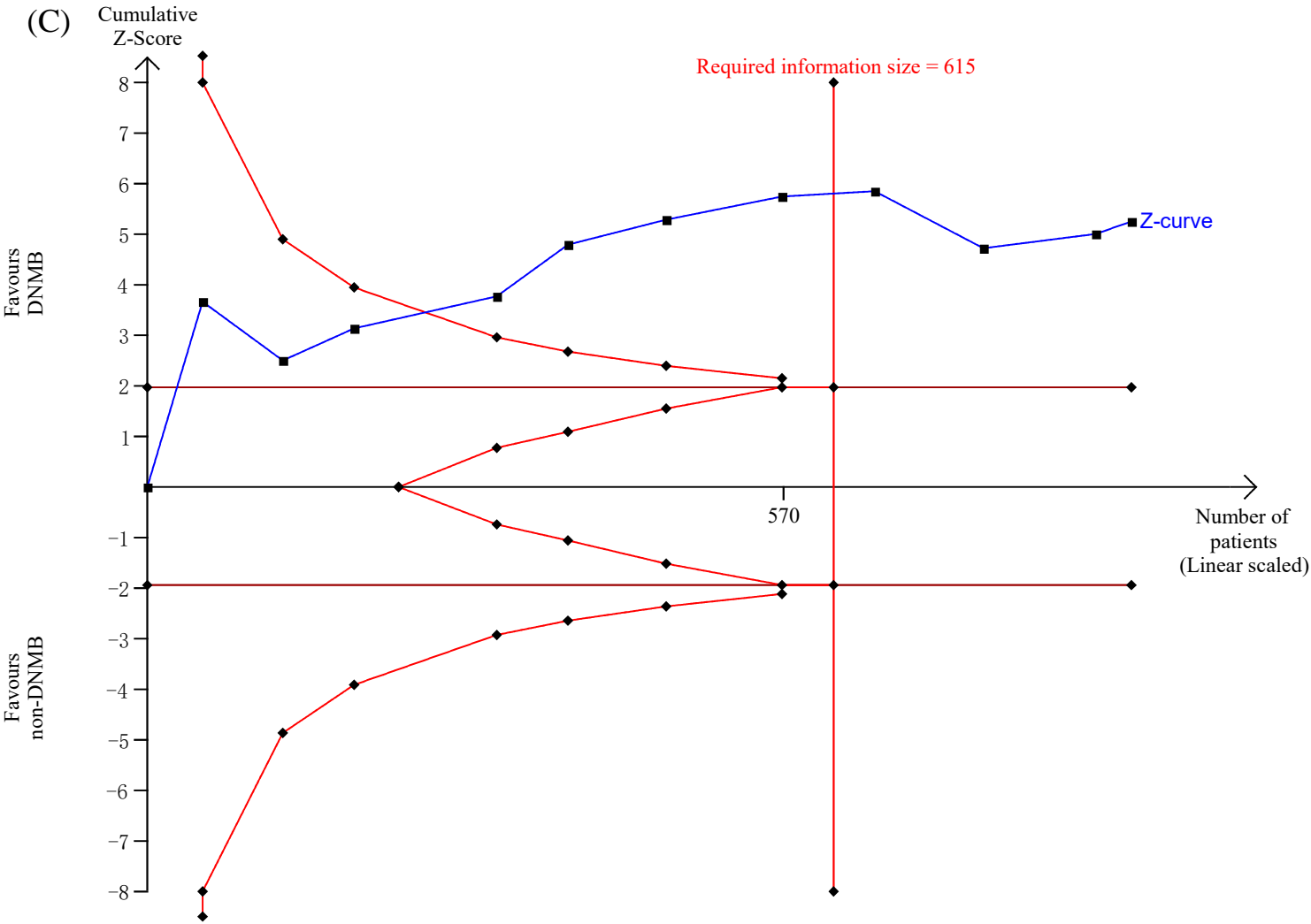

Supplement: S11 Fig — (A) Acceptable surgical condition, (B) Surgical condition score, (C) Intraoperative movement. (PDF) [file pone.0282790.s011.pdf]
