# Supplementary material for: Does deep neuromuscular blockade provide improved perioperative outcomes in adult patients? A systematic review and meta-analysis of randomized controlled trials
Source: PLoS One. 2023 Mar 9;18(3):e0282790. doi: 10.1371/journal.pone.0282790 (PMC9997990; doi:10.1371/journal.pone.0282790)
Supplement: S1 Table — (DOCX) [file pone.0282790.s012.docx]

#### S1 Table. Search strategies for PubMed, Embase, the Cochrane Central Register of Controlled Trials (CENTRAL), and Google scholar.

| Database | Item | Search strategies | N |
| --- | --- | --- | --- |
| PubMed | #1 | ("Neuromuscular blockade"[MeSH Terms] OR neuromusc*[tiab]) | 66086 |
|  | #2 | (Depth[tiab] OR Deep[tiab] OR profound[tiab] OR intense[tiab] OR extreme[tiab] OR moderate[tiab] OR middle[tiab] OR medium[tiab] OR middle[tiab] OR shallow[tiab] OR low[tiab]) | 4479469 |
|  | #3 | (((randomized controlled trial or controlled clinical trial).pt. or randomi*ed.ab. or placebo.ab. or drug therapy.fs. or randomly.ab. or trial.ab. or groups.ab.) not (exp animals/ not humans.sh.)) | 6177852 |
|  | #4 | #3 AND (#1 AND #2) | 2697 |
| Embase | #1 | 'neuromuscular blockade'/exp OR 'neuromuscular blockade' OR 'neuromusc*':ab,ti | 90119 |
|  | #2 | 'deep':ab,ti OR 'profound':ab,ti OR 'intense':ab,ti OR 'extreme':ab,ti OR 'moderate':ab,ti OR 'medium':ab,ti OR 'middle':ab,ti OR 'shallow':ab,ti OR 'low':ab,ti | 5478239 |
|  | #3 | #2 AND #3 AND [randomized controlled trial]/lim | 946 |
| CENTRAL | #1 | Neuromuscular blockade AND neuromusc* | 2114 |
|  | #2 | Depth OR Deep OR profound OR intense OR extreme OR moderate OR middle OR medium OR middle OR shallow OR low | 658135 |
|  | #3 | #1 AND #2 | 1232 |
| Google scholar | #1 | Neuromuscular blockade AND neuromusc* | 1310 |
|  | #2 | Depth OR Deep OR profound OR intense OR extreme OR moderate OR middle OR medium OR middle OR shallow OR low | 8580000 |
|  | #3 | #1 AND #2 | 1160 |
