# Supplementary material for: Does deep neuromuscular blockade provide improved perioperative outcomes in adult patients? A systematic review and meta-analysis of randomized controlled trials
Source: PLoS One. 2023 Mar 9;18(3):e0282790. doi: 10.1371/journal.pone.0282790 (PMC9997990; doi:10.1371/journal.pone.0282790)
Supplement: S2 Table — (DOCX) [file pone.0282790.s013.docx]

#### S2 Table. Sensitivity analysis.

| **Outcome** | **N of studies** | **N of patients** | **Heterogeneity** | |  | **Pooled results** |  |
| --- | --- | --- | --- | --- | --- | --- | --- |
|  |  |  | **I^2^** | ***p*** |  | **MD/RR** | ***p*** |
| **Excluding studies at high risk of bias** | | | | | | | |
| Acceptable surgical condition | 19 | 1719 | 57% | 0.001 |  | 1.19 (1.11, 1.28) | <0.00001 |
| Surgical condition score | 24 | 1978 | 86% | <0.00001 |  | 0.51 (0.34, 0.68) | <0.00001 |
| Intraoperative movement | 6 | 525 | 49% | 0.08 |  | 0.17 (0.07, 0.42) | 0.0001 |
| Additional measure to improve surgical condition | 8 | 559 | 61% | 0.01 |  | 0.55 (0.32, 0.93) | 0.03 |
| Intraoperative blood loss | 3 | 212 | 47% | 0.15 |  | -45.36 (-115.56, 24.84) | 0.21 |
| Duration of surgery | 27 | 2307 | 32% | 0.06 |  | 0.07 (-2.27, 2.41) | 0.95 |
| Pain at 24 h | 5 | 300 | 60% | 0.04 |  | -0.18 (-0.43, 0.08) | 0.17 |
| Pain at 48 h | 3 | 166 | 50% | 0.13 |  | -0.57 (-1.26, 0.13) | 0.11 |
| Length of stay | 10 | 990 | 4% | 0.41 |  | -0.13 (-0.28, 0.02) | 0.10 |
|  |  |  |  |  |  |  |  |
| **Converting to fixed effect model** | | | | | | | |
| Acceptable surgical condition | 22 | 1984 | 55% | 0.001 |  | 1.13 (1.09, 1.18) | <0.00001 |
| Surgical condition score | 28 | 2276 | 85% | <0.00001 |  | 0.35 (0.30, 0.41) | <0.00001 |
| Intraoperative movement | 11 | 883 | 24% | 0.21 |  | 0.30 (0.21, 0.43) | <0.00001 |
| Additional measure to improve surgical condition | 11 | 817 | 66% | 0.001 |  | 0.75 (0.62, 0.90) | 0.002 |
| Intraoperative blood loss | 5 | 329 | 51% | 0.09 |  | -9.07 (-17.52, -0.61) | 0.04 |
| Duration of surgery | 35 | 2937 | 28% | 0.07 |  | 1.12 (0.70, 1.55) | <0.00001 |
| Pain at 24 h | 10 | 691 | 87% | <0.00001 |  | -0.31 (-0.35, -0.27) | <0.00001 |
| Pain at 48 h | 7 | 457 | 86% | <0.00001 |  | -0.49 (-0.67, -0.31) | <0.00001 |
| Length of stay | 14 | 1312 | 28% | 0.15 |  | -0.00 (-0.01, 0.01) | 0.96 |
