# Supplementary material for: Does deep neuromuscular blockade provide improved perioperative outcomes in adult patients? A systematic review and meta-analysis of randomized controlled trials
Source: PLoS One. 2023 Mar 9;18(3):e0282790. doi: 10.1371/journal.pone.0282790 (PMC9997990; doi:10.1371/journal.pone.0282790)
Supplement: S3 Table — (PDF) [file pone.0282790.s014.pdf]

Certainty assessment							№ of patients		Effect		Certainty	Importance
**№ of studies**	**Study design**	**Risk of bias**	**Inconsistency**	**Indirectness**	**Imprecision**	**Other considerations**	**DNMB**	**Non-DNMB**	**Relative (95% CI)**	**Absolute (95% CI)**		
**Acceptable surgical conditions**												
22	randomised trials	serious[a]	not serious	not serious	not serious	publication bias strongly suspected dose response gradient[b]	832/993 (83.8%)	692/991 (69.8%)	**RR 1.19** (1.11 to 1.27)	**133 more per 1,000** (from 77 more to 189 more)	⊕⊕⊕◯ Moderate	CRITICAL
**Surgical condition score**												
28	randomised trials	serious[a]	serious[c]	not serious	not serious	publication bias strongly suspected dose response gradient[b]	1141	1135	-	MD **0.52 SD higher** (0.37 higher to 0.67 higher)	⊕⊕◯◯ Low	CRITICAL
**Intraoperative movement**												
11	randomised trials	serious[a]	not serious	not serious	not serious	publication bias strongly suspected very strong association dose response gradient[b]	24/445 (5.4%)	122/438 (27.9%)	**RR 0.19** (0.10 to 0.33)	**226 fewer per 1,000** (from 251 fewer to 187 fewer)	⊕⊕⊕⊕ High	CRITICAL
**Additional measures to improve surgical condition**												
11	randomised trials	serious[a]	serious[c]	not serious	not serious	publication bias strongly suspected dose response gradient[b]	104/406 (25.6%)	169/411 (41.1%)	**RR 0.63** (0.43 to 0.94)	**152 fewer per 1,000** (from 234 fewer to 25 fewer)	⊕⊕◯◯ Low	CRITICAL
**Intraoperative blood loss**												
5	randomised trials	serious[a]	not serious	not serious	not serious	publication bias strongly suspected[b]	165	164	-	MD **22.8 lower** (48.83 lower to 3.24 higher)	⊕⊕◯◯ Low	CRITICAL
**Duration of surgery**												
35	randomised trials	serious[a]	not serious	not serious	not serious	none	1470	1467	-	MD **0.05 lower** (2.05 lower to 1.95 higher)	⊕⊕⊕◯ Moderate	CRITICAL
**Pain at 24 h**												
10	randomised trials	serious[a]	serious[c]	not serious	serious[d]	none	345	346	-	MD **0.42 SD lower** (0.74 lower to 0.1 lower)	⊕◯◯◯ Very low	CRITICAL
**Pain at 48 h**												
7	randomised trials	serious[a]	serious[c]	not serious	serious[d]	none	229	228	-	MD **0.49 SD lower** (1.03 lower to 0.05 higher)	⊕◯◯◯ Very low	CRITICAL
**Length of stay**												
14	randomised trials	serious[a]	not serious	not serious	not serious	publication bias strongly suspected[b]	653	659	-	MD **0.05 lower** (0.19 lower to 0.08 higher)	⊕⊕◯◯ Low	CRITICAL

CI: confidence interval; **MD:** mean difference; **RR:** risk ratio

Explanations

a. The proportion of information from studies at some concern or high risk of bias is likely to be sufficient to affect the interpretation of results.
b. The asymmetry of the funnel plot is suggestive of publication bias.
c. Considerable heterogeneity.
d. Criteria for an optimal information size are not met.
