# Supplementary material for: Does deep neuromuscular blockade provide improved perioperative outcomes in adult patients? A systematic review and meta-analysis of randomized controlled trials
Source: PLoS One. 2023 Mar 9;18(3):e0282790. doi: 10.1371/journal.pone.0282790 (PMC9997990; doi:10.1371/journal.pone.0282790)
Supplement: Attachment — Submitted filename: Response to Reviewers.docx [file pone.0282790.s016.docx]

Dear editors and reviewers:

Thank you for your letter and comments on our manuscript titled “Does deep neuromuscular blockade provide improved perioperative outcomes in adult patients? A systematic review and meta-analysis of randomized controlled trials” (PONE-D-22-20092). These comments helped us improve our manuscript, and provided important guidance for future research.

We have addressed the editor’s and the reviewers’ comments to the best of our abilities and revised the text to meet the PLOS ONE style requirements. We hope this meets your requirements for a publication. Relevant revisions made in response to reviewer comments, we marked the revised portions in red in the manuscript. The main comments and our specific response are detailed below:

**Academic Editor:**

**Please add confidence intervals to the pooled surgical score.**

Response: Thanks for your suggestion, we have added confidence intervals to the pooled surgical score (Lines 265-267 in the manuscript).

**Editors:**

**1. Please ensure that your manuscript meets PLOS ONE's style requirements, including those for file naming.**

Response: Thanks for your suggestion, we have made the corresponding changes according to the submission guidelines of PLOS ONE. We have uploaded all the figure files to the Preflight Analysis and Conversion Engine (PACE) digital diagnostic tool and passed the certification. So, we re-uploaded our figure files.

**2. Upon re-submitting your revised manuscript, please upload your study’s minimal underlying data set as either Supporting Information files or to a stable, public repository and include the relevant URLs, DOIs, or accession numbers within your revised cover letter.**

Response: This study is a systematic review, and all data are derived from published articles, which can be found in the manuscript and supporting information files. Our study protocol was published in the International Prospective Register of Systematic Reviews. Changed Data Availability Statement is below: A pre-specified study protocol was published in the International Prospective Register of Systematic Reviews (PROSPERO) database (CRD42022301248, https://www.crd.york.ac.uk/PROSPERO/display_record.php?RecordID=301248). All relevant data are within the manuscript and its Supporting information files.

**Reviewer 1:**

**1. Methods section: the difference between "acceptable surgical condition" and "surgical condition score" is a bit confusing. The Results and Discussion sections help to understand the difference, but the definitions in M&M need to be clear. Perhaps it would be useful to add a figure showing the surgical scale that underlines which score the authors have defined as "acceptable".**

Response: We are sorry that this part was not clear in the original manuscript. We have added a paragraph describing definitions of outcome indicators (Lines 117-139 in the manuscript). Considering a large number of figures in the manuscript, we have not added new figures and only used words to describe the definition of "acceptable surgical condition" and "surgical condition score" (Lines 117-129 in the manuscript).

**2. Methods section: Similarly it is not clear what are the "measures to improve the surgical condition". Please provide more information (although the Discussion section provides some definitions).**

Response: Thanks for your suggestion, we have added a paragraph describing definitions of "additional measures to improve the surgical condition" (Lines 132-135 in the manuscript).

**3. Results section: If possible, it would be interesting to have some data on what "measures to improve the surgical condition" have been taken.**

Response: Thanks for your suggestion, we have added new sentences describing specific measures to improve the surgical condition, their references, and their applicable conditions (Lines 309-315 in the manuscript).

**4. Discussion Section: The authors have included many types of surgeries and I agree with them that this is an important strength of their work. However, they should point out that non-laparoscopic studies are few and very heterogeneous (only 9 studies including about 700 patients). Therefore, it is true that the overall effect size is important, but the subgroup analysis is weak for non-laparoscopic studies. Large differences in optimal surgical conditions are generally expected between orthopedics, ENT surgeons, and interventional radiologists. it is difficult to target these needs and this is one of the reasons for the need for further investigation in the future.**

Response: Thanks for your affirmation and suggestion on our work. We acknowledge that the type of surgery is one of the major sources of heterogeneity. Statistically, both a small sample size and high heterogeneity suggest low evidence quality. In clinical practice, different surgeries have different requirements for the neuromuscular blockade, and different surgeries have different manifestations of deterioration of surgical workspace conditions. In the different types of surgeries, the measures to improve the surgical condition are different. Therefore, we have added a paragraph to explain this issue, and we have stated that this is one of the limitations of this paper and that this is one of the reasons for the need for further investigation (Lines 450-470 in the manuscript).

**5. I have some concern about complications analysis. It is very difficult to evaluate the protective effect of NMBA reversal (both neostigmine and sugammadex). This issue is beyond the scope of the present study but it should be stated by the authors that they did not take into account in their analysis this important aspect of neuromuscular blockade management.**

Response: Thanks for your suggestion. According to our pre-specified study protocol, residual neuromuscular blockade should have been one of the adverse events. We cited references to illustrate the incidence of residual neuromuscular blockade (both neostigmine and sugammadex) and its adverse consequences in the original manuscript, to demonstrate the importance and rationality of residual neuromuscular blockade as an adverse event. Unfortunately, all included studies did not report this important outcome, so the question of whether DNMB increases the incidence of residual neuromuscular blockade is beyond the scope of our systematic review. According to your suggestion, we have changed this paragraph to state this issue (Lines 541-553 in the manuscript).

**Reviewer 2:**

**The authors need to redo their analysis and include only studies in which Deep block was indeed compared to moderate (or shallow block). This requires that not only the deep blockade is maintained by infusion, guided by neuromuscular monitoring, but that the moderate (or shallow) block is also maintained by infusion, guided by neuromuscular monitoring. Without this approach, it is not possible to conclude that deep block is superior to moderate block.**

Response: Thanks for your suggestion. The details of the revised manuscript and responses are as follows:

(1) Before re-analyses, we have re-screened all the included studies and have found that 2 included studies have a fluctuating level of neuromuscular blockade in the control arm due to their cross-over design (Madsen 2015-REF 47, Söderström 2018-REF 52 in the original manuscript) and 8 included studies have no specific level of neuromuscular blockade in the control arm (REF 13, 25, 29, 46, 47, 48, 50, 51 in the revised manuscript). Based on the pre-specified study protocol, we should only include parallel randomized control trials, so we have removed two cross-over studies that have a fluctuating level of neuromuscular blockade in the control arm (Madsen 2015-REF 47, Söderström 2018-REF 52 in the original manuscript).

(2) According to our pre-specified study protocol (CRD42022301248), the intervention is the deep neuromuscular block and the comparator is the shallower neuromuscular block (from Moderate block to acceptable recovery). We are sorry that we used the unclear word “MNMB/SNMB group” to describe the shallower neuromuscular block. We have changed this issue and used “DNMB group” and “non-DNMB group” to describe the interventions. (Lines 106-107 in the manuscript).

(3) We do not think it is meaningless to include the shallower blockade than MNMB (including shallow block, minimal block, and acceptable recovery) in the control arm. Although, from your point, “in the study by Dubois et al (REF 29) Fig 3 clearly reports that surgical conditions were unsatisfactory when the block was minimal”, Dubois et al only included participants receiving laparoscopic surgery and did not report other outcomes (including intraoperative movement, and adverse events et al.). As we all know, different surgeries have different requirements for the level of neuromuscular blockade, while orthopedic surgeons, otolaryngologists, and interventional radiologists often differ greatly in their expectations of optimal surgical conditions. The loose inclusion criteria are one of the main strengths of this systematic review, designed to increase the [applicability](javascript:;) of the conclusions.

In the revised manuscript, we have reserved 8 included studies with no specific level of neuromuscular blockade in the control arm (REF 13, 25, 29, 46, 47, 48, 50, 51 in the manuscript), and we have used “non-MNMB” to describe the constantly changing level of neuromuscular blockade. To adequately and rigorously compare DNMB and MNMB, we have divided studies into two subgroups (the DNMB vs MNMB subgroup and the DNMB vs non-MNMB subgroup). In the DNMB vs non-MNMB subgroup, moderate neuromuscular blockade was maintained and guided by neuromuscular monitoring. After re-analyses, we found that the main conclusions had not changed. Considering the large sample size in the DNMB vs MNMB subgroup, the analyses in the DNMB vs MNMB subgroup are stable. We have changed the paragraph in the discussion part to adequately and rigorously explain this issue (Lines 471-490 in the manuscript).

(4) According to the results of re-analyses, we have revised all figures, tables, and supporting information files.

We would be glad to respond to any further questions and comments that you may have.

Sincerely yours,

Wang Xiong
