# Supplementary material for: Does deep neuromuscular blockade provide improved perioperative outcomes in adult patients? A systematic review and meta-analysis of randomized controlled trials
Source: PLoS One. 2023 Mar 9;18(3):e0282790. doi: 10.1371/journal.pone.0282790 (PMC9997990; doi:10.1371/journal.pone.0282790)
Supplement: Attachment — Submitted filename: Response to Reviewers.docx [file pone.0282790.s017.docx]

Dear editors and reviewers:

Thank you for your comments and suggestions on our manuscript titled “Does deep neuromuscular blockade provide improved perioperative outcomes in adult patients? A systematic review and meta-analysis of randomized controlled trials” (PONE-D-22-20092R1). These helped us improve our manuscript.

We have addressed the issues to the best of our abilities and marked the revised portions in red in the manuscript. We hope these meet your requirements for a publication. The main comments and our specific response are detailed below:

**Editors:**

**1. However, it is extremely important to emphasize the significant limitations in the existing literature. Systematic reviews are limited by the quality of the individual trials analyzed and reviewed. What may be viewed as “minor” variations in methodology may markedly affect outcome. Some frequently cited studies are often poorly designed to give us the answers we really need.**

Response: Thanks for your comment. We agree with you. Different articles are bound to differ in methodology. The main role of a systematic review is to comprehensively explore the similarities and differences between different articles and cautiously draw conclusions. In this systematic review, the quality of each article was evaluated by the risk of bias assessment (lines 238-245 in the Revised Manuscript with Track Changes), and the quality of evidence for each conclusion was evaluated accordingly by the Grades of Recommendation, Assessment, Development and Evaluation (GRADE) system (lines 406-413). Consequentially, conclusions based on higher-quality articles have higher evidence quality. According to the limitations of existing articles, this systematic review puts forward suggestions for improvement in the conclusion part, which is conducive to improving the quality of future research. We believe that with the improvement of the quality of the research design, the quality of conclusions will be improved.

**2. The magnitude of industry involvement needs to be transparently disclosed, please add data on which studies were supported by Merck, this can be done with a simple table.**

Response: Thanks for your suggestion. We had a statement about this limitation (lines 571-574). In reference to your suggestion, we have added the funding source item in Table 1.

**3. The manuscript is importantly improved with the separation of comparative studies in two categories, DNMB and non-DNMB, respectively. However, it should be stated as a limitation that we do not know in several studies how well controlled the moderate block was. Was it indeed consistently a moderate block?**

Response: Thanks for your comment. In the primary analysis, we divided the interventions into two groups (DNMB versus non-DNMB). Non-DNMB is the more superficial level of a neuromuscular blockade than DNMB, including moderate neuromuscular blockade, shallow block, and minimal block (lines 61-66). There was a limitation that the neuromuscular blockade management protocols are different among studies, and some studies did not provide the details of the depth of neuromuscular blockade or did not use neuromuscular monitoring to maintain a specific depth of neuromuscular blockade (Table 1). To address this issue, we divided the non-DNMB group into two subgroups including DNMB vs MNMB subgroup and the DNMB vs non-MNMB subgroup (lines 182-186). We have stated this limitation and suggested future studies should maintain a specific depth of neuromuscular blockade by neuromuscular monitoring and report the details of the neuromuscular blockade management protocol (lines 494-500).

**4. Your manuscript cites a review by Richebe which is generally supportive of the concept of DNMB. I suggest you mention that the topic is controversial and you may want to cite a critical review, too. You may consider Naguib and Kopmans critical review of the topic (Anesth Analg 2015;120:51–8).**

Response: Thanks for your suggestion. We have added a statement (lines 56-57) and cited the critical review you mentioned to emphasize that the topic is controversial (Ref 3).

**5. Your manuscript also does not discuss a different and potentially clinically important conclusion which is that** **DNMB may be important only in a subset of patients. It is possible that this subset of patients could be identified by improved intraoperative communication between surgeon and anesthesiologist.**

Response: Thanks for your comment. We agree with you. Our systematic review attempted to address this issue. In the study protocol, we hypothesized that different types of surgery may have an effect on the outcome, which is one of the important strengths of our manuscript compared with other systematic reviews (line 183). In the subgroup analysis, we divided the type of surgery into multiple subgroups, including laparoscopic surgery, laparotomy, orthopedic surgery, thoracoscopic surgery, laryngeal surgery, urologic surgery, and interventional radiography. The results showed that the outcome was stable in the laparoscopic surgery subgroup. In other surgical subgroups, the heterogeneity was high, and the sample size was small, so the outcome was unstable. We think that we draw a reasonable conclusion based on existing articles. Future studies with larger sample sizes and different types of surgery are needed to test the hypothesis that DNMB may be important only in a subset of patients. We had a paragraph in the discussion section to emphasize this limitation (lines 473-475).

**6. One important aspect is whether opportunities are sought to lower the pneumoperitoneal pressure. How often is this done? Should future studies address this? Many institutions and surgeons use the same pressure for all patients, is this rational? All of these issues amount to shortcomings in the current literature and they limit what we can conclude from systematic reviews.**

Response: Thanks for your comment. We think the problem you mentioned is worth exploring. There is no unified consensus or guideline on how to manage pneumoperitoneum pressure, and the management plan of each hospital is not the same. Whether DNMB can improve perioperative outcomes and its mechanism remain unclear, and one of the mechanisms may be DNMB allowing lower pneumoperitoneum pressure. Based on the existing studies, we cannot solve the questions you mentioned, and these questions are not able to be answered by this systematic review. Considering the important value of the questions you mentioned, we think it is necessary to emphasize this limitation of current studies in the discussion section. So, we added a statement. We expect more and more researchers to pay attention to these issues (lines 522-528) and hope that these issues can be solved in future studies and systematic reviews.

We would be glad to respond to any further questions and comments that you may have.

Sincerely yours,

Wang Xiong
